# Crosstalk between the calcineurin and cell wall integrity pathways prevents chitin overexpression in *Candida albicans*

**DOI:** 10.1242/jcs.258889

**Published:** 2021-12-16

**Authors:** Alessandra da Silva Dantas, Filomena Nogueira, Keunsook K. Lee, Louise A. Walker, Matt Edmondson, Alexandra C. Brand, Megan D. Lenardon, Neil A. R. Gow

**Affiliations:** 1 School of Biosciences, University of Exeter, Geoffrey Pope Building, Exeter EX4 4QD, UK; 2 School of Medical Sciences, Institute of Medical Sciences, University of Aberdeen, Aberdeen AB25 2ZD, UK; 3 Children's Cancer Research Institute, Labdia and Max F. Perutz Laboratories, Vienna 1090, Austria; 4 NGeneBio Company, 288 Digital-ro, Guro-gu, Seoul 08390, South Korea; 5 School of Biotechnology and Biomolecular Sciences, University of New South Wales, Sydney, NSW 2052, Australia

**Keywords:** Antifungal drug, Ca^2+^-calcineurin, Chitin, Caspofungin, Cell wall stress, Echinocandins

## Abstract

Echinocandins such as caspofungin are frontline antifungal drugs that compromise β-1,3 glucan synthesis in the cell wall. Recent reports have shown that fungal cells can resist killing by caspofungin by upregulation of chitin synthesis, thereby sustaining cell wall integrity (CWI). When echinocandins are removed, the chitin content of cells quickly returns to basal levels, suggesting that there is a fitness cost associated with having elevated levels of chitin in the cell wall. We show here that simultaneous activation of the calcineurin and CWI pathways generates a subpopulation of *Candida albicans* yeast cells that have supra-normal chitin levels interspersed throughout the inner and outer cell wall, and that these cells are non-viable, perhaps due to loss of wall elasticity required for cell expansion and growth. Mutations in the Ca^2+^-calcineurin pathway prevented the formation of these non-viable supra-high chitin cells by negatively regulating chitin synthesis driven by the CWI pathway. The Ca^2+^-calcineurin pathway may therefore act as an attenuator that prevents the overproduction of chitin by coordinating both chitin upregulation and negative regulation of the CWI signaling pathway.

This article has an associated First Person interview with the first author of the paper.

## Abstract (precis)

**Summary:** The pathogenic fungus *Candida* can limit the overproduction of chitin in its cell wall to prevent loss of fitness via a calcineurin-dependent mechanism.

## INTRODUCTION

Echinocandins are a major class of frontline antifungal drugs that result in damage to the essential cell wall polysaccharide β-1,3 glucan, thereby exhibiting fungicidal effects against *Candida* species, *Aspergillus fumigatus* and other fungal pathogens (Walker et al., 2010; Mroczyńska and Brillowska-Dąbrowska, 2020). Most fungi have two structural polysaccharides in the cell wall – β-1,3 glucan and chitin – and both contribute to its overall strength (Gow et al., 2017). The structure of chitin makes it one of the strongest biomaterials in nature. It is a homopolymer of β-1,4 *N*-acetyl-glucosamine that exists as non-elastic, antiparallel-folded, linear hydrogen-bonded chains that form robust microfibrils in the cell wall (Gow and Gooday, 1983; Gow et al., 1987; Lenardon et al., 2010). Fungi can strengthen their cell wall when β-1,3 glucan is damaged by elevating the chitin content to levels that compensate for glucan damage and maintain cell viability (Lee et al., 2012, 2018; Yang et al., 2017; Ries et al., 2017). It is therefore important to understand the mechanism by which echinocandin exposure leads to modifications of the cell wall.

The incidence of mucosal and systemic *Candida* infections has increased in recent years, with life-threatening invasive candidiasis reaching 2-14 annual cases per 100,000 of the population and 6-9 cases per 1000 patients in intensive care units (Sanguinetti et al., 2015; Kullberg and Arendrup, 2015; Pappas et al., 2018). There is a global annual burden of ∼250,000 invasive, life-threatening *Candida* infections and more than 100 million recurrent mucosal vaginitis (Kullberg and Arendrup, 2015; Denning et al., 2018).

Intravenous caspofungin is now used routinely in the treatment of systemic fungal infection as first-line choice of drug for the treatment of *Candida* species (Patil and Majumdar, 2017; Perlin, 2020). Caspofungin non-competitively inhibits Fks1, the catalytic subunit of β-1,3 glucan synthase, preventing the *de novo* formation of β-1,3 glucan, thereby compromising the assembly of the cantilevered glucan-chitin wall exoskeleton (Hoang, 2001; Steinbach and Perfect, 2003; Denning, 2003; Odds et al., 2003; Walker et al., 2010; Földi et al., 2012; Lenardon et al., 2020). The resulting disturbance in cell wall architecture compromises cell wall integrity (CWI), leading to cell swelling and ultimately to lysis. Although the number of *Candida albicans* strains that are resistant to echinocandins is low (2-3%), an increasing number of reports of *Candida* breakthrough infections after prolonged therapy have been published (Shields et al., 2015a; Perlin, 2015; Perlin et al., 2017). In addition, in the emerging pathogen *Candida auris*, echinocandin resistance occurs in 30% of all isolates from some parts of the world (Kordalewska et al., 2018; Arastehfar et al., 2020). Point mutations in two major hotspots of the *FKS1* gene are normally associated with increased resistance to echinocandins both *in vitro* and *in vivo* (Park et al., 2005; Katiyar et al., 2006; Lee et al., 2012; Astvad et al., 2013; Shields et al., 2015a; Perlin et al., 2017; Pfaller et al., 2019). However, not all cases of breakthrough infections during echinocandin treatment are associated with *FKS1* mutations (Drakulovski et al., 2011; Shields et al., 2015b; Healey et al., 2017; Perlin et al., 2017; Fraser et al., 2020).

Fungal cells can resist the damaging effects of echinocandins by upregulation of chitin synthesis (Walker et al., 2008; Fortwendel et al., 2010; Ben-Ami et al., 2011; Yang et al., 2013; Walker et al., 2015; Yang et al., 2017). The increase in total chitin content seen in caspofungin-tolerant *C. albicans* cells is coordinated simultaneously by cell wall integrity, Ca^2+^-calcineurin and high osmolarity glycerol (HOG) pathways (Munro et al., 2007). This redundancy of mechanisms means that genetic deletions of single key regulators of these pathways (e.g. *mkc1*Δ, *cna1*Δ, *cnb1*Δ and *hog1*Δ) do not prevent cells from being able to increase their chitin content in response to caspofungin. Both calcineurin and the CWI pathways are positively regulated by Ca^2+^ influx. Increases in Ca^2+^ concentration results in the formation of a Ca^2+^-calmodulin complex that de-phosphorylates its targets (Cruz et al., 2002). In addition, Ca^2+^ influx via the Cch1-Mid1 channel also activates the CWI pathway in other fungi (Mishra et al., 2017). Furthermore, previous reports have suggested cross-talk may exist between the Ca^2+^-calcineurin and the CWI pathway (Munro et al., 2007; LaFayette et al., 2010; Colabardini et al., 2014; Donlin et al., 2014; Altwasser et al., 2015; Tanaka et al., 2018; Jiang et al., 2018; Pianalto et al., 2019).

Fungal cells with an elevated chitin content generally display increased tolerance to caspofungin. Some strains of *Candida* with high-chitin levels exhibit ‘paradoxical growth’, defined as enhanced growth of the organism at caspofungin concentrations above the minimum inhibitory concentration (MIC) (Stevens et al., 2006; Wiederhold, 2009; Melo et al., 2007; Bizerra et al., 2011; Shields et al., 2011; Rueda et al., 2014). However, high-chitin cells grown under the environmental selective pressure of an echinocandin quickly revert to generating progeny with lower chitin levels in their walls when the echinocandin is removed. This implies that there is a fitness cost in having a cell wall with elevated chitin (Ben-Ami and Kontoyiannis, 2012).

The aim of this work was to understand the mechanism by which upregulation of chitin is maintained and regulated in response to cell wall stress. We show that some cells that die in the presence of caspofungin had supra-high chitin levels, whereas cells that survive caspofungin treatment have moderate levels of chitin in their wall. This supports the hypothesis that having too much chitin in the wall represents a fitness and viability cost. Multiple mechanisms must be induced simultaneously in order to generate supra-high chitin cells. We propose that a mechanism exists to normally maintain chitin levels below a threshold concentration and that when this mechanism is compromised, further increases in chitin content begin to compromise cell viability. We demonstrate that the Ca^2+^-calcineurin pathway is essential for this chitin synthesis attenuating mechanism that prevents overproduction of chitin by negatively regulating the CWI signaling pathway (Fig. S1).

## RESULTS

### Yeast cells with supra-high chitin content lose viability

**Fig. 1.**
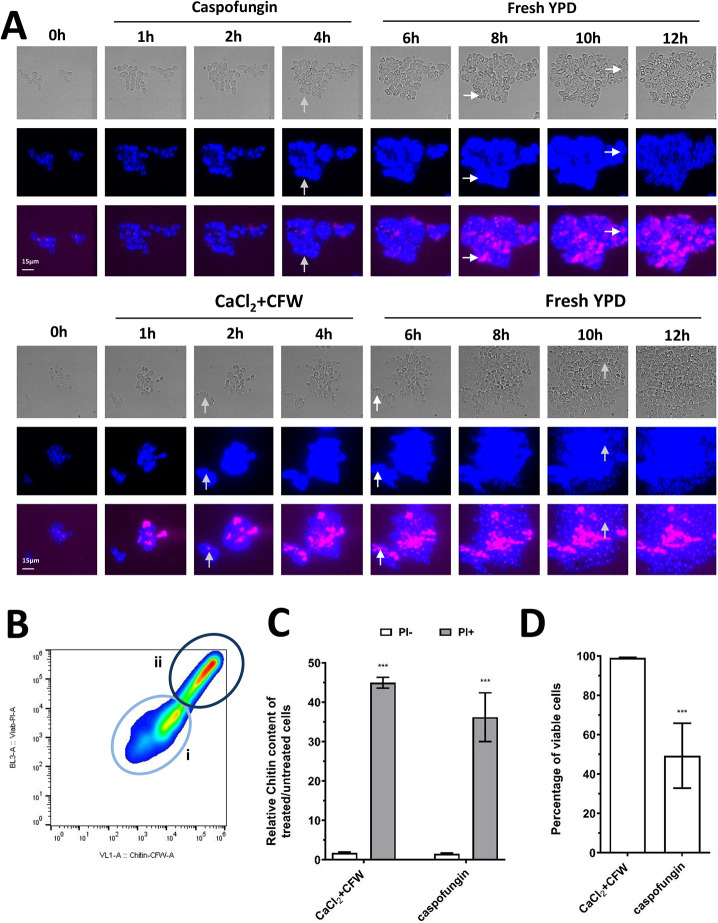
**Supra-high chitin cells associated with loss of viability.** (A) Representative micrographs of microfluidics analyses where *C. albicans* wild-type cells were treated with either 0.2 M CaCl_2_+100 µg/ml CFW or 2 µg/ml caspofungin, followed by removal of these treatments and continued growth on YPD. Total chitin was visualized using CFW staining (blue) and PI staining (magenta). Gray arrows indicate viable high chitin cells; white arrows indicate non-viable high chitin cells. (B) FACS plot representative of an experiment where *C. albicans* wild-type cells were treated with 2 µg/ml caspofungin for 6 h. Two subpopulations are present: low PI/CFW fluorescence that represents live cells (light-blue circle, i) and high PI/CFW fluorescence that represents dead cells (dark-blue circle, ii). (C) Ratio of chitin content of live (PI negative) and growth arrested/dead yeast cells (PI positive). Values are representative of the median fluorescence intensity (MFI) ratio±s.e.m. of the chitin content between 0.2 M CaCl_2_+100 µg/ml CFW treated/untreated cells or 2 µg/ml caspofungin treated/untreated cells (*n*≥2). (D) Percentage of viable cells following treatment with either 0.2 M CaCl_2_+100 µg/ml CFW or 2 µg/ml caspofungin for 12 h. Scale bars: 15 µm. ***P≤0.05 (one-way ANOVA test with Dunnett's post-hoc *t*-test).

The survival of *C. albicans* treated with cell wall damaging agents has been shown to be promoted by increasing the chitin content of their cell wall (Walker et al., 2008, 2015; Fortwendel et al., 2010; Ben-Ami et al., 2011; Yang et al., 2013, 2017). Using microfluidic imaging, we observed that treatment of *C. albicans* with CaCl_2_ and Calcofluor White (CFW; referred to hereafter as CaCl_2_+CFW) or with caspofungin induced substantial increases in chitin content relative to untreated controls, as assessed visually by the intensity of CFW fluorescence (Fig. 1A; 0 h versus 1-12 h time points). CaCl_2_+CFW or caspofungin treatment resulted in the formation of cells that had visibly increased chitin formation (Fig. 1A, gray and white arrows), including a sub-set of non-viable cells displaying high levels of chitin in the wall (Fig. 1A, white arrows). The number of high chitin PI-positive cells increased progressively in the presence of caspofungin, but these cells did not form high chitin buds and underwent limited cell expansion. These high chitin cells could only form buds (with a normal chitin content) after caspofungin had been removed (Fig. 1A, upper panel, white arrows, 6-12 h, Movies 1 and 2). PI positivity increased progressively in cells that had high chitin content (Fig. 1A, Figs S2 and S3). In contrast, treatment with CaCl_2_+CFW resulted in high chitin cells that retained the ability to replicate during and after CaCl_2_+CFW exposure, and predominantly remained PI negative (Fig. 1A, lower panel, gray arrows, 6-12 h). Furthermore, while high-chitin mother cells remained growth arrested or died in the presence of cell wall stressors, daughter cells were able to replicate and filament when the stressor was removed (Fig. S2, Movies 1 and 2). These observations suggest that CaCl_2_+CFW treatment increased chitin to levels that were tolerable and did not significantly affect viability, whereas the very high chitin levels seen in caspofungin-treated cells were harmful, causing growth arrest, loss of viability and ultimately cell death. Combinations of both CaCl_2_+CFW and caspofungin further accelerated the loss of cell viability (data not shown).

Next, we assessed these characteristics quantitatively by growing *C. albicans* yeast cells in the presence or absence of either CaCl_2_+CFW or caspofungin for 12 h and used fluorescence-activated cell sorting (FACS) to measure the relative chitin content (CFW median fluorescence intensity of treated cells compared with untreated cells) in live (PI negative) and dead (PI positive) cells. Cell populations with the highest CFW staining correlated with a higher proportion of PI-positive cells (Fig. 1B,C). Whereas live cells displayed an increase in median CFW fluorescence intensity (MFI) of approximately twofold compared with untreated cells, dead cells had supra-high chitin levels in their wall, in which the median fluorescence intensity was increased by more than 30-fold relative to untreated cells (Fig. 1C). Both live and dead cells were observed following treatment with CaCl_2_+CFW or caspofungin. However, only 2% of cells were PI positive following treatment with CaCl_2_+CFW alone, whereas 50% of cells were PI positive following treatment with caspofungin (Fig. 1D). Irrespective of the method of inducing chitin synthesis, cells with the highest chitin contents were non-viable (Fig. 1B,C). Differences in viability between CaCl_2_+CFW and caspofungin-treated cells could be due to the extent of wall elasticity as a function of chitin content. Chitin is a non-elastic polysaccharide, whereas β-1,3 glucan has a degree of elasticity due to its tri-helical structure. We therefore examined the ratio of β-1,3 glucan and chitin in cells treated with either CaCl_2_+CFW or caspofungin (Fig. S4). This confirmed that there was a significant increase in the proportion of chitin relative to β-1,3 glucan in the cell walls of both CaCl_2_+CFW (0.2%) and caspofungin-treated (1.5%) cells compared with untreated *C. albicans* (Fig. S4). These data suggest that cells with supra-high chitin levels became non-viable, which we hypothesize is due the lack of capacity for cell expansion in a non-elastic cell wall.

### Treatment with CaCl_2_+CFW activates a mechanism that prevents the overexpression of chitin synthesis in response to caspofungin

In FACS analyses, the majority of cells (∼98%) treated with CaCl_2_+CFW were viable and did not have supra-high levels of chitin (Fig. 1B-D). In contrast, 50% of caspofungin-treated cells were PI positive (dead) with supra-high chitin levels (Fig. 1B-D). We hypothesized that treatment of *C. albicans* with CaCl_2_+CFW for 12 h partially activated chitin synthesis and that this treatment did not lead to overexpression of chitin to the extent that non-viable supra-high chitin cells were formed. Therefore, cells treated with CaCl_2_+CFW had moderate levels of chitin and 98% of yeast cells remained viable. In contrast, treatment with caspofungin strongly stimulated the overexpression of chitin and induced the formation of supra-high chitin cells that resulted in PI positivity in at least 50% of yeast cells (Fig. 1D).

**Fig. 2.**
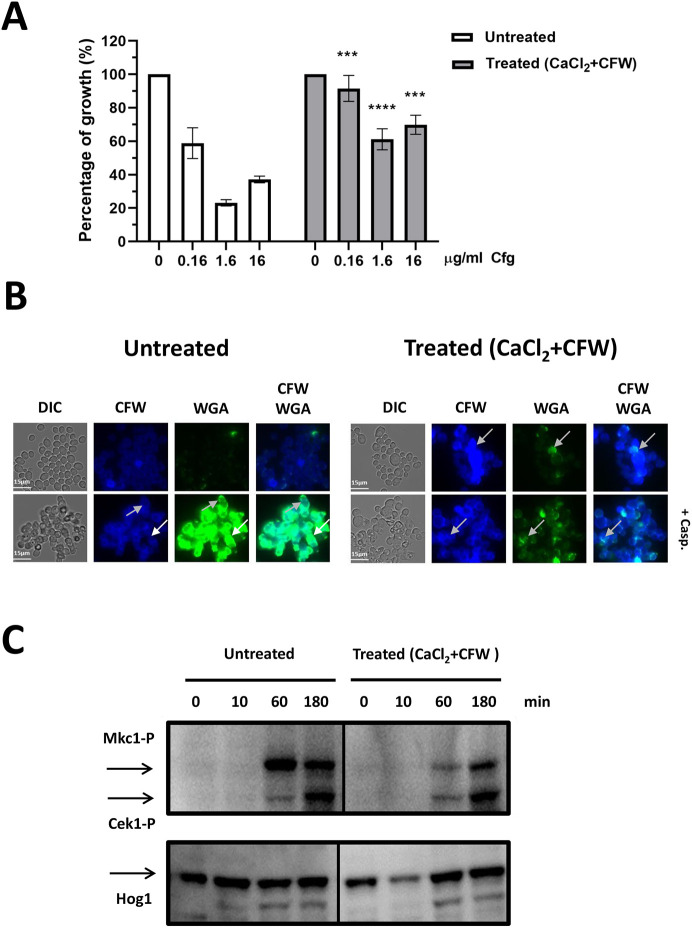
**Pre-treatment of cells with CaCl_2_+CFW attenuates caspofungin-induced Mkc1 activation.** Cells were grown in the absence or presence of 0.2 M CaCl_2_+100 µg/ml CFW for 12 h with and without caspofungin. (A) Percentage growth following 24 h incubation in the presence of caspofungin (Cfg) at the indicated doses comparing cells pre-treated with CaCl_2_+CFW with cells without pre-treatment at each caspofungin concentration. ***P≤0.05, ****P≤0.01 (one-way ANOVA test with Dunnett's post-hoc *t*-test). (B) DIC and fluorescence images of untreated or CaCl_2_+CFW pre-treated cells grown in the absence or presence of caspofungin (Casp; 2 µg/ml) for 6 h. Total chitin was visualized by CFW staining and exposed chitin by staining with WGA. White arrows indicate supra-high chitin cells, where chitin is accessible to WGA; gray arrows represent moderate to high chitin cells, where chitin is less accessible to WGA. (C) Mkc1 (Mkc1-P) and Cek1 (Cek1-P) phosphorylation determined by western blotting analysis of lysates prepared from untreated or Ca^2+^+CFW pre-treated wild-type cells grown in the absence or presence of caspofungin (2 µg/ml). Hog1 levels were used as loading controls. Scale bars: 15 µm.

We then tested whether cells that had been treated with CaCl_2_+CFW could subsequently be induced to overexpress chitin by subsequent treatment with caspofungin. We compared the susceptibility of cells to different concentrations of caspofungin for 24 h with and without pre-treatment with CaCl_2_+CFW (Fig. 2A). We found that pre-treatment of cells with CaCl_2_+CFW increased growth in the presence of caspofungin by 1.5-3 fold (*P*≤0.005) (Fig. 2A). This suggested that pre-treatment with CaCl_2_+CFW may inhibit a mechanism that elevated chitin to supra-high levels in the presence of caspofungin.

We then assessed where chitin was deposited in the cell wall in response to treatment with CaCl_2_+CFW and caspofungin. We stained cells with CFW, which freely penetrates the inner and outer cell wall layers, as an indicator of total chitin (Fig. 2B, CFW), and with the high molecular weight lectin wheat germ agglutinin (WGA), which cannot normally access the inner cell wall layer and therefore only binds surface-exposed chitin (Fig. 2B, WGA) (Mora-Montes et al., 2011). Pre-treatment with CaCl_2_+CFW moderately increased the amount of chitin in cells (Fig. 2B, top panels, CFW gray arrows), but did not result in exposure of chitin at the surface (Fig. 2B, top panels, WGA). In contrast, treatment with caspofungin resulted in intense WGA staining, indicating a significant increase in surface-exposed chitin (Fig. 2B, bottom panels, WGA, white arrows). However, this was reduced when cells were pre-treated with CaCl_2_+CFW (Fig. 2B, bottom panels, WGA). Therefore, CaCl_2_+CFW pre-treatment of cells reduced both the amount and the surface exposure of chitin relative to treatment with caspofungin alone. This suggests that CaCl_2_+CFW activated a mechanism that limited or attenuated both chitin overexpression and exposure of chitin in the outer cell wall.

Phosphorylation of the mitogen-activated protein kinase (MAPK) Mkc1 was assessed to determine whether the activation of this CaCl_2_+CFW-dependent attenuation mechanism involved the CWI pathway in cells that had been pre-treated with CaCl_2_+CFW and then exposed to caspofungin for 10 min, 60 min or 3 h. Cells treated with caspofungin only were used as the control (Fig. 2C). Activated Mkc1 was detected in both non-treated and CaCl_2_+CFW pre-treated cells after 60 min treatment with caspofungin but the level of phosphorylated Mkc1 in pre-treated cells was significantly reduced at this time point. This was also the case at 180 min, although to a lesser extent. Phosphorylation of Cek1 (an ERK family protein kinase involved in an unrelated signaling pathway) was unchanged between non-pre-treated and pre-treated cells. Therefore, activation of the CWI pathway on treatment with caspofungin was lower in cells that had been pre-treated with CaCl_2_+CFW. This suggests that a CaCl_2_+CFW-dependent mechanism may downregulate the CWI pathway and limit the incorporation of cell wall chitin that would otherwise have been stimulated by caspofungin. Together, these results suggest that supra-high chitin levels in the wall of *C. albicans* positively correlates with the generation of PI-positive cells and loss of viability, and that a CaCl_2_+CFW-dependent mechanism downregulates chitin synthesis. This mechanism may normally prevent chitin overexpression to levels that compromise cell viability. Activation of this chitin attenuation mechanism, which negatively regulates the CWI pathway, results in cells that remained viable in the presence of caspofungin by preventing hyper-activation of chitin synthesis (Fig. S1).

### The Ca^2+^-calcineurin pathway prevents chitin overexpression

Wild-type cells grown in the presence of CaCl_2_+CFW activate a mechanism that prevents the formation of supra-high chitin cells by negatively regulating the CWI pathway (above). Full induction of chitin synthase (*CHS*) gene expression in cells treated with CaCl_2_+CFW involves the Ca^2+^-calcineurin, HOG and CWI pathways (Munro et al., 2007). To further explore the role of these signaling pathways on this attenuation mechanism, FACS was used to screen key mutants in these pathways for the loss of their ability to prevent generation of supra-high chitin cells. The mutants that were tested included: *cek1*Δ, *cek2*Δ and *cph1*Δ (members of the Cek1 MAPK pathway); *hog1*Δ, *rck2*Δ, *msn4*Δ and *sko1*Δ (HOG pathway); *pkc1*Δ, *mkc1*Δ, *swi4*Δ and *swi6*Δ (additional members of the CWI pathway); *cna1*Δ, *cnb1*Δ and *crz1*Δ (Ca^2+^-calcineurin pathway); and *tpk1*Δ, *tpk2*Δ and *efg1*Δ (cAMP pathway).

**Fig. 3.**
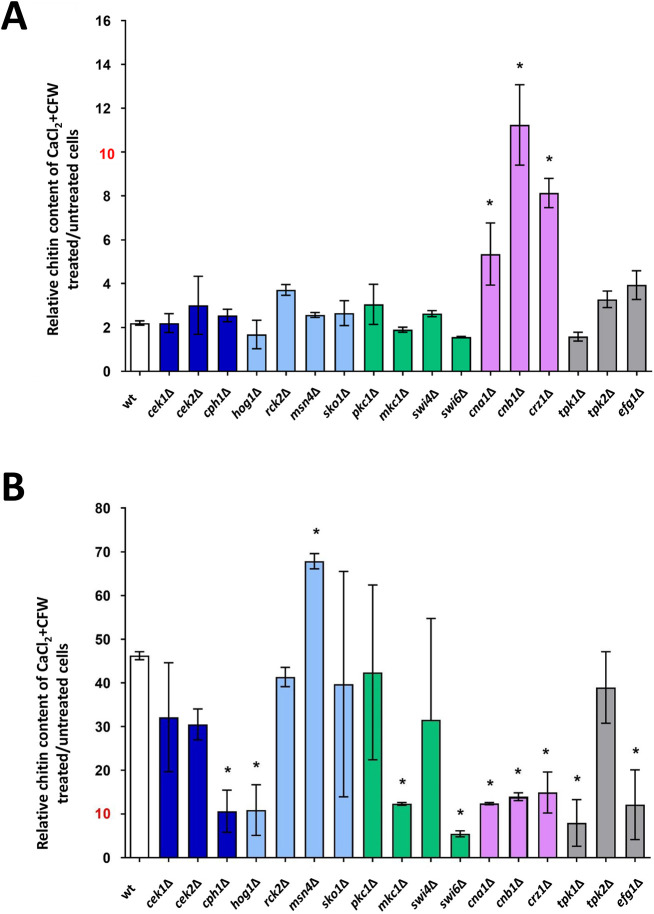
**Inhibition of the Ca^2+^-calcineurin pathway is associated with appearance of viable supra-high chitin cells.** Cells were grown in the absence or presence of 0.2 M CaCl_2_+100 µg/ml CFW for 12 h, and the relative chitin content in live (PI negative, A) and dead cells (PI positive, B) was measured. Data are shown for wild-type cells (wt) and cells lacking key regulators or components of the following pathways: Cek1 mitogen-activated protein kinase signaling pathway (*cek1*Δ and *cph1*Δ, dark-blue bars); Cek2 mitogen-activated protein kinase signaling pathway (*cek2*Δ and *cph1*Δ, dark-blue bars); Hog1 mitogen-activated protein kinase signaling pathway (*hog1*Δ, *rck2*Δ, *msn4*Δ and *sko1*Δ, light-blue bars); Mkc1 mitogen-activated protein kinase signaling pathway (*pkc1*Δ, *mkc1*Δ, *swi4*Δ and *swi6*Δ, green bars); Ca^2+^- calmodulin-calcineurin (Ca^2+^-CAM) signaling pathway (*cna1*Δ, *cnb1*Δ and *crz1*Δ, light-purple bars); PKA signaling pathway (*tpk1*Δ, *tpk2*Δ and *efg1*Δ, gray bars). Data are the median fluorescence intensity (MFI) ratio±s.e.m., which is representative of the chitin content of CaCl_2_+CFW pre-treated/untreated cells in at least two experimental replicates (*n*=2-5). *P≤0.05 (one-way ANOVA test with Dunnett's post-hoc *t*-test).

Wild-type and mutant cells were grown in the presence or absence of CaCl_2_+CFW for 12 h and FACS was used to measure the relative chitin content through the median fluorescence intensity (CFW MFI) of treated cells compared with untreated cells in live (PI negative) and dead (PI positive) cells. In wild-type cells, treatment with CaCl_2_+CFW resulted in an approximate doubling of MFI of PI-negative cells (Fig. 3A). Mutations in the calcineurin pathway had a significantly elevated chitin content relative to wild-type control cells (Fig. 3A), suggesting that, under these conditions, this pathway negatively regulates chitin synthesis. The chitin content of PI-positive cells with supra-high chitin content exhibited a ∼30-50-fold increase in MFI (Fig. 3B).

Live *C. albicans* mutants lacking key regulators of the Ca^2+^-calcineurin pathway (*cna1*Δ, *cnb1*Δ and *crz1*Δ) had significantly higher MFI values following CaCl_2_+CFW treatment than wild-type cells (*P*≤0.05), strongly suggesting a role for this pathway in downregulating chitin synthesis (Fig. 3A, light purple). The six- to 12-fold increase in relative chitin content observed by MFI in these mutants (compared with a twofold increase in wild-type cells) did not result in the loss of viability. These mutants retained the ability to replicate and were PI negative (Fig. 1A, lower panel, light-gray arrows and Fig. 3). An additional member of the CWI pathway (Swi6) was also identified as playing a role in the chitin attenuation mechanism. The *swi6*Δ mutant also had a significantly higher MFI value (*P*≤0.05) (Fig. 3A). Therefore, the Ca^2+^-calcineurin pathway stands out in this analysis as playing a key role in attenuating excessive chitin synthesis.

We also assessed whether these mutants were impacted in their ability to form cells with supra-high chitin levels. Mutants that are unable to induce supra-high chitin cells are likely to represent genes that are involved in promoting chitin synthesis following cell wall stress, whereas mutants that are able to form supra-high chitin cells are likely to represent genes that are dispensable for synthesis of supra-high chitin cells. Dead cells with supra-high chitin levels were observed in wild-type cells and some mutants (Fig. 3B). However, dead cells with supra-high chitin content were not observed in mutants in the Cek1 MAPK (*cph1*Δ), Hog1 MAPK (*hog1*Δ), cell wall salvage (*mkc1*Δ and *swi6*Δ), Ca^2+^-calcineurin (*cna1*Δ, *cnb1*Δ and *crz1*Δ) or cAMP (*tpk1*Δ and *efg1*Δ) pathways (Fig. 3B). Furthermore, the *msn4*Δ mutant displayed significant higher levels of chitin (∼1.5 fold) in its wall compared with wild-type cells, indicating a role for Msn4 in inhibiting chitin synthesis. Therefore, multiple pathways are involved in the generation of non-viable supra-high chitin cells (Fig. S1).

### Calcium channels and upstream regulators of the Ca^2+^-calcineurin pathway are dispensable for the chitin attenuator mechanism

**Fig. 4.**
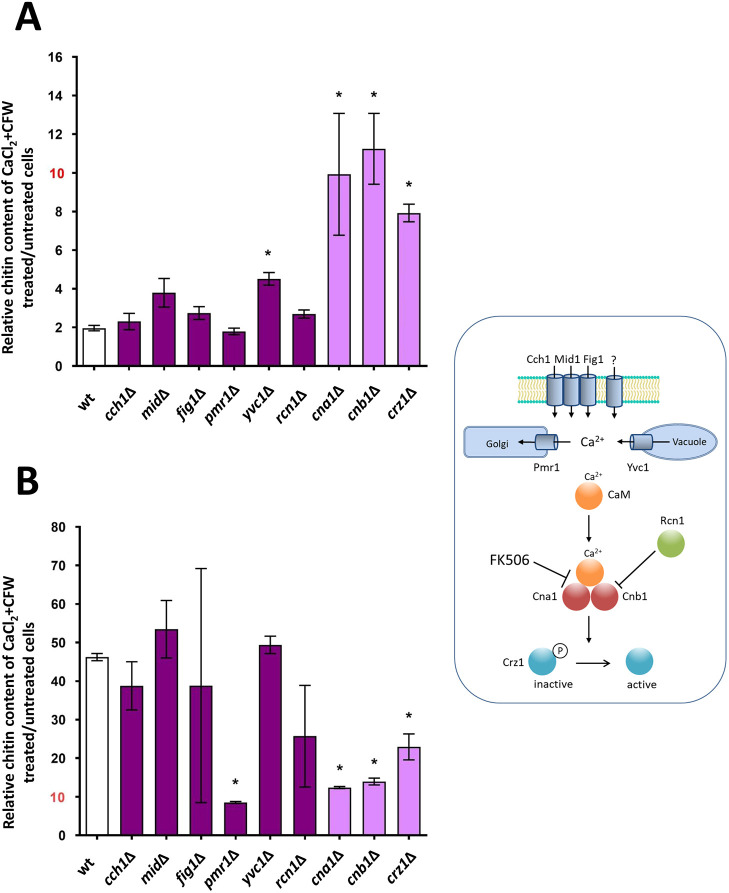
**Effect of mutations in the Ca^2+^-calcineurin pathway on chitin content.** Chitin content of yeast cells following Ca^2+^+CFW treatment in mutants of three plasma membrane Ca^2+^ influx facilitators (Mid1, Cch1 and Fig1), the vacuolar Ca^2+^ efflux channel Yvc1, the Golgi Ca^2+^ ATPase Pmr1, and the Rcn1 negative regulator of the Ca^2+^-calcineurin pathway (shown on the right). Cells were grown in the absence or presence of 0.2 M CaCl_2_+100 µg/ml CFW for 12 h and the ratio of chitin content of live (PI negative, A) and dead cells (PI positive, B) measured in wild-type cells (wt) and in mutant strains. Data for *cna1*Δ, *cnb1*Δ and *crz1*Δ (lighter colored bars) are from the same mutant screen (Fig. 3). Values are the median fluorescence intensity (MFI) ratio±s.e.m., which represents the chitin content between CaCl_2_+CFW pre-treated/untreated cells in at least two experimental replicas (*n*=2-5). *P≤0.05 (one-way ANOVA test with Dunnett's post-hoc *t*-test).

Our results indicated that the Ca^2+^-calcineurin pathway plays a key role in preventing excessive chitin synthesis. We therefore expanded our analysis from three mutants of this pathway (*cna1*Δ, *cnb1*Δ and *crz1*Δ) to include all members of this pathway, starting with the sensors and other potential regulators of the pathway (Fig. 4). *C. albicans* has at least three plasma membrane-localized Ca^2+^-influx facilitators, Mid1, Cch1 and Fig1, that positively regulate calcineurin activity (Brand et al., 2007; Yang et al., 2011). We hypothesized that stretch-activation of Ca^2+^ influx may act as a sensor of changes in cell wall plasticity, which may be influenced by chitin content. Similarly, Pmr1 has the potential to regulate cell wall plasticity in response to CaCl_2_+CFW, because this ATPase links transport of Ca^2+^ into the Golgi with glycosylation of cell wall proteins, which are required for protecting *C. albicans* against cell wall stress (Bates et al., 2005). Furthermore, the Yvc1 vacuolar Ca^2+^ channel (Yu et al., 2014) and the predicted negative regulator Rcn1 (Reedy et al., 2010) inhibit calcineurin function; therefore, the deletion mutants were tested for their ability to regulate chitin production.

The MFI of mutants deleted for *CCH1*, *MID1*, *FIG1*, *PMR1* or *RCN1* did not show the same increase in relative chitin content that was observed in *cna1*Δ, *cnb1*Δ and *crz1*Δ cells following CaCl_2_+CFW treatment (Fig. 4A), suggesting that they are not necessary for the chitin attenuation mechanism. We also observed that dead cells with supra-high chitin levels were formed in all mutants except for the *pmr1*Δ mutant, indicating that the Golgi Ca^2+^ transporter, Pmr1, contributed to induction of supra-high chitin cells (Fig. 4B).

**Fig. 5.**
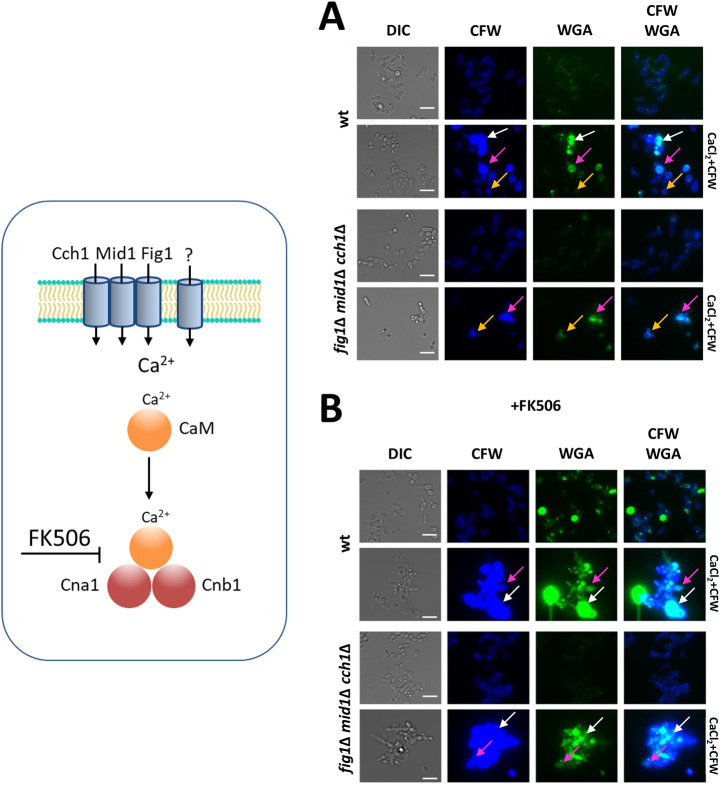
**Chitin exposure at the cell surface requires calcineurin but not the Fig1, Mid1 or Cch1 Ca^2+^-influx facilitators.** Putative Ca^2+^ channels and components of the calcineurin pathway are shown on the left. DIC and fluorescence images of cells grown in the absence (A) or presence of 12.5 µg/ml FK506 (12 h) (B), followed by treatment with CaCl_2_+CFW for 6 h. Total chitin was visualized using CFW and exposed chitin in the outer cell wall was stained with WGA. White arrows indicate supra-high chitin cells; magenta arrows indicate moderate to high chitin cells; orange arrows indicate high chitin cells. Scale bars: 15 μm.

In addition, the total amount of chitin (CFW staining) and surface-exposed chitin (WGA staining) of a triple mutant lacking all three calcium-influx facilitators was not affected when assessed using CFW and WGA staining, with or without treatment with CaCl_2_+CFW (Fig. 5A). This confirmed that Ca^2+^ influx was not essential for the activation of the chitin attenuation mechanism under these conditions. In addition, treatment of the *cch1*Δ/*mig1*Δ/*fig1*Δ triple mutant with calcineurin inhibitor FK506 and CaCl_2_+CFW still resulted in moderate increases in relative chitin content, indicating that channel activity was dispensable for the activation of chitin upregulation (Fig. 5B). Collectively, these results suggest that activation of the chitin attenuator mechanism that prevents supra-high chitin formation is not dependent on individual upstream regulators of the Ca^2+^-calcineurin pathway in *C. albicans* (Fig. S1).

### Crosstalk between the Ca^2+^-calcineurin and CWI pathways regulates Chs2 and Chs3

**Fig. 6.**
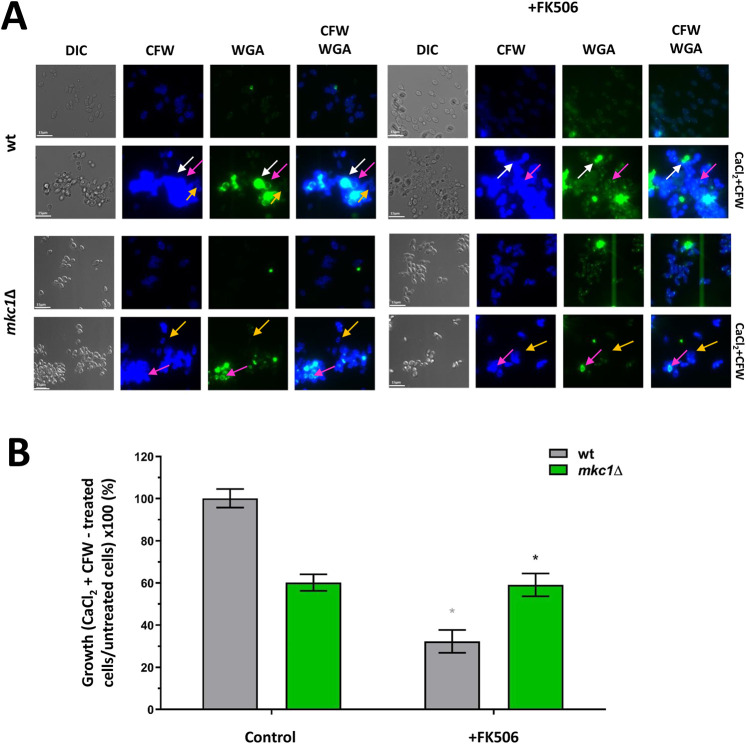
**Interaction between the calcineurin and Mkc1 cell integrity pathways in controlling chitin production.** (A) DIC and fluorescence images of cells grown in the absence (left), or presence (right) of the calcineurin inhibitor FK506, for 12 h, followed by treatment with CaCl_2_+CFW for 6 h. Chitin was visualized by staining with CFW (total chitin) and WGA (exposed chitin). White arrows indicate supra-high chitin cells; magenta arrows indicate moderate to high chitin cells; orange arrows indicate high chitin cells. Scale bars: 15 μm. (B) *C. albicans* cells grown with or without FK506 for 12 h, followed by incubation in either YPD or YPD+CaCl_2_+CFW for 24 h. Controls contained DMSO diluent alone. Results are expressed as growth percentage of cells incubated in YPD with CaCl_2_+CFW compared with cells incubated in YPD without CaCl_2_+CFW. Error bars indicate s.e.m. from three experimental replicates (*n*=3). *P≤0.05 (one-way ANOVA test with Dunnett's post-hoc *t*-test).

Cna1, Cnb1 and Crz1, and the CWI pathway are required to prevent the formation of supra-high chitin levels in the cell wall. We hypothesized, therefore, that calcineurin may regulate the CWI pathway to prevent moderate to high chitin levels from accumulating in stressed cells. This was assessed by comparing the total amount of chitin (CFW staining) and surface-exposed chitin (WGA staining) of *mkc1*Δ mutant cells grown in the presence or absence of the calcineurin inhibitor FK506, followed by treatment with CaCl_2_+CFW (Fig. 6A).

Again, we observed that both total chitin and exposed chitin increased in CaCl_2_+CFW-treated wild-type cells, irrespective of the presence of FK506 (Fig. 6A). The *mkc1*Δ mutant had reduced chitin levels following CaCl_2_+CFW, but this did not impact chitin exposure in the outer layers of the wall (Fig. 6A, left panel). On the other hand, when grown in the presence of FK506 and CaCl_2_+CFW, *mkc1*Δ cells were unable to increase their chitin content and a significant reduction in both total and exposed chitin levels was observed (Fig. 6A, right panel). Thus, the CWI pathway is required to induce moderate to high levels of chitin in cells where the Ca^2+^-calcineurin pathway was inhibited.

The *mkc1*Δ null mutant displayed similar levels of growth when grown in the presence or absence of FK506 (Fig. 6B). This genetic evidence supports the hypothesis that the CWI pathway is central to this calcineurin-mediated effect on viability following cell wall stress. Furthermore, we observed a 60% reduction in growth (OD_600_ for 24 h) of wild-type cells that were pre-grown in the presence of FK506 before being exposed to CaCl_2_+CFW (Fig. 6B). Deletion of *MKC1* therefore partially mitigated growth inhibition associated with CaCl_2_+CFW treatments, when grown in the presence of the calcineurin inhibitor (+FK506) (Fig. 6B). These results again support a role for the calcineurin pathway in negatively regulating the CWI pathway and chitin synthesis, hence promoting cell viability.

**Fig. 7.**
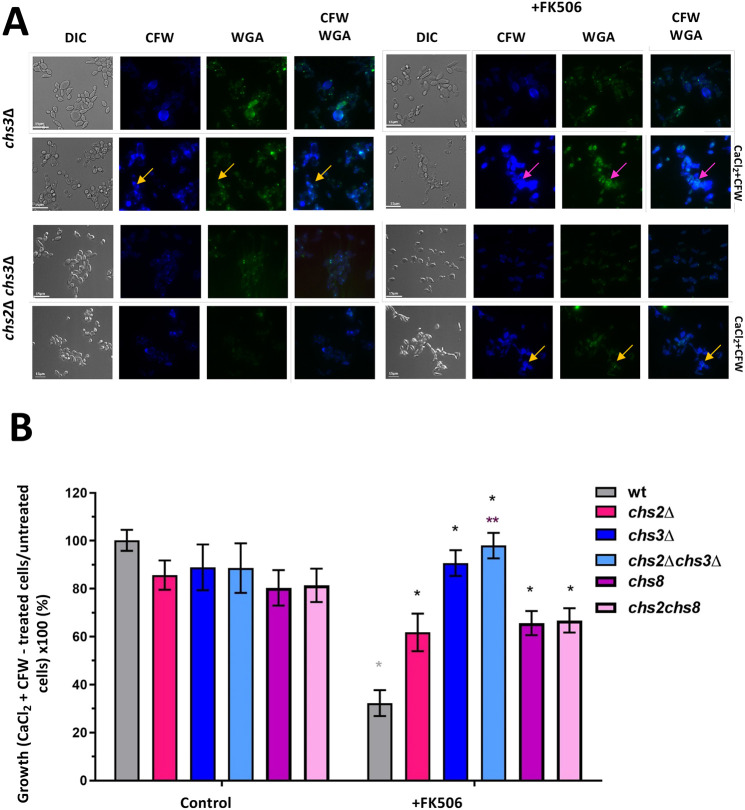
**Deletion of Chs2 and Chs3 rescues cell growth in the presence of CaCl_2_+CFW when calcineurin is inhibited by FK506.** (A) DIC and fluorescence images were taken of *C. albicans* cells grown in the absence (left) or presence (right) of the calcineurin inhibitor FK506, for 12 h, followed by treatment with CaCl_2_+CFW for 6 h. Chitin was visualized using CFW (total) and WGA (exposed) staining. Scale bars: 15 μm. Magenta arrows indicate moderate to high chitin cells; orange arrows indicate high chitin cells. (B) *C. albicans* cells were grown with or without FK506 for 12 h, followed by incubation in either YPD or YPD+CaCl_2_+CFW for 24 h. The control contained DMSO diluent. Results are expressed as the growth of cells incubated in YPD with CaCl_2_+CFW as a percentage of cells grown in YPD without CaCl_2_+CFW. Error bars indicate s.e.m. from three experimental replicates (*n*=3). *P≤0.05; **P≤0.005 (one-way ANOVA test with Dunnett's post-hoc *t*-test).

In *C. albicans*, chitin is synthesized by four Chs isoenzymes: Chs1, Chs2, Chs3 and Chs8 (Lenardon et al., 2010). CaCl_2_+CFW treatment upregulates all four *CHS* genes at the level of transcription (Munro et al., 2007) but upregulation of *CHS2* is specifically dependent on Mkc1 (Munro et al., 2007). In addition, *chs3*Δ mutant cells display reduced chitin levels following CaCl_2_+CFW treatment compared with untreated cells (Munro et al., 2007). Therefore, we stained *chs3*Δ and *chs2*Δ*chs3*Δ cells with CFW and WGA after growth in the presence or absence of FK506 followed by treatment with CaCl_2_+CFW and examined them by fluorescence microscopy (Fig. 7A). The *chs3*Δ mutant was compromised in its ability to increase cell chitin content following treatment with CaCl_2_+CFW (Fig. 7A), which is consistent with the results obtained by Munro et al. (2007). The generation of moderate to high chitin cells also required Chs3, as *chs3*Δ cells treated with FK506 formed few moderate to high chitin cells following CaCl_2_+CFW stimulation (Fig. 7A). There was no significant increase in chitin content of the double *chs2*Δ*/chs3*Δ mutant following CaCl_2_+CFW treatment, and these cells failed to form moderately high and supra-high chitin levels in cells in the presence of FK506 and CaCl_2_+CFW (Fig. 7A).

Furthermore, mutants in *CHS2*, and to a greater extent *CHS3*, were mitigated in the growth inhibition associated with FK506 treatment (Fig. 7B). This supports the hypothesis that calcineurin negatively regulates chitin synthesis, thereby promoting viability by preventing accumulation of supra-high levels of chitin in the wall. We conclude that calcineurin negatively regulates activation of the CWI pathway and chitin synthesis mediated by Chs2 and Chs3, and consequently the viability is preserved (Fig. S1A).

### Chitin localization and Mkc1 activation after caspofungin treatment

**Fig. 8.**
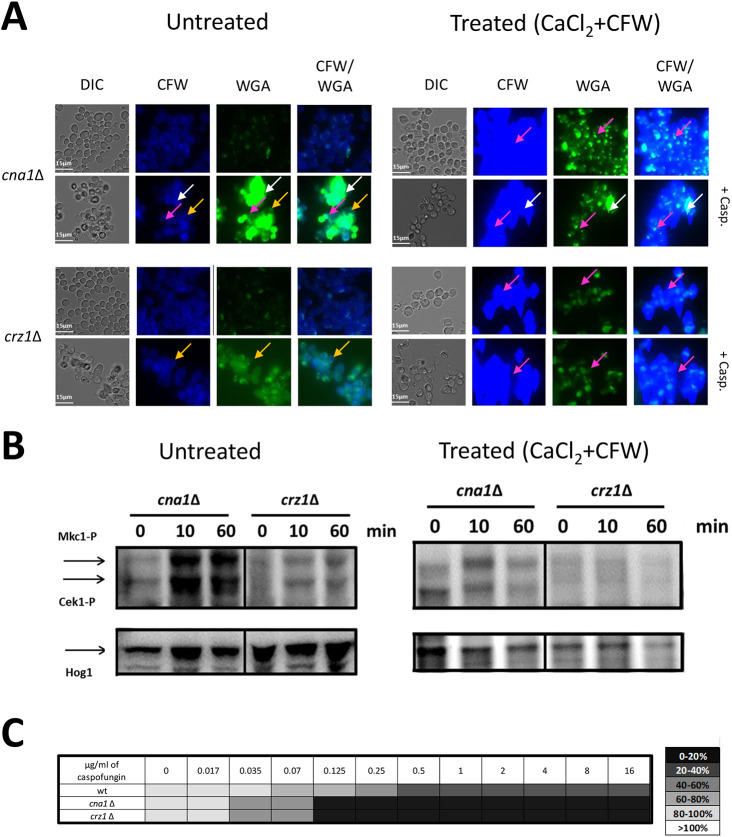
**Calcineurin modulates chitin synthesis and activation of the cell salvage pathway following caspofungin treatment.** (A) DIC and fluorescence images of CaCl_2_+CFW pre-treated/untreated cells grown in the absence or presence of caspofungin (Casp; 2 µg/ml). Chitin of *cna1*Δ and *crz1*Δ mutants was visualized using CFW for total cell wall staining, and WGA to display exposed chitin. White arrows indicate supra-high chitin cells; magenta arrows indicate moderate to high chitin cells; orange arrows indicate high chitin cells. Scale bars: 15 μm. (B) Mkc1 (Mkc1-P) and Cek1 (Cek1-P) phosphorylation determined by western blotting analysis of lysates prepared from CaCl_2_+CFW-treated or untreated cells grown in the presence of caspofungin (2 µg/ml). Hog1 levels were used as loading controls. (C) Susceptibility to caspofungin of Ca^2+^-calcineurin mutants. *C. albicans* cells were grown in YPD for 12 h, washed in water and inoculated in YPD containing increasing concentrations of caspofungin. OD_600_ was measured after 24 h of incubation at 30°C. Results are reported as percentage of viability in a heat map format.

We assessed the effect of caspofungin treatment on the activation of the cell integrity pathway in *cna1*Δ and *crz1*Δ mutants. In the absence of pre-treatment with CaCl_2_+CFW, caspofungin treatment of wild-type and *cna1*Δ *C. albicans* resulted in the formation of supra-high chitin cells, in which chitin was located primarily exposed in outer layers of the wall (Figs 2B and 8A). This contrasts with treatment of *cna1*Δ mutants with caspofungin after CaCl_2_+CFW pre-treatment, which resulted in a subpopulation of cells that contained high chitin levels in both the inner and outer cell wall (Fig. 8A). In *crz1*Δ cells, irrespective of CaCl_2_+CFW pre-treatment, caspofungin caused augmented chitin levels, but this was located in both the inner and the WGA-accessible outer layers of the wall. Therefore, few supra-high chitin cells were formed and there was less WGA staining (Fig. 8A).

Caspofungin treatment induced activation of the CWI pathway in wild-type cells (Fig. 2C). Quicker and stronger activation of Mkc1 was seen in caspofungin-treated *cna1*Δ cells, and reduced Mkc1 phosphorylation was observed in the *crz1*Δ null mutant (Fig. 8B). These results support the hypothesis that Cna1 and Crz1 negatively regulate the CWI pathway under conditions where high levels of chitin synthesis is induced. Pre-treatment of cells with CaCl_2_+CFW prevented supra-high chitin accumulation and reduced Mkc1 phosphorylation that would otherwise be stimulated by exposure to caspofungin (Figs 2B and 8B). These results suggest that upregulation of chitin associated with CaCl_2_+CFW pre-treatment could result in dampening of signaling via the CWI pathway and suggest the presence of other regulators of this pathway following CaCl_2_+CFW pre-treatment. Supporting this, the calcineurin pathway mutants *cna1*Δ, *cnb1*Δ and *crz1*Δ all showed increased chitin content compared with wild type in response to CaCl_2_+CFW and a significantly increased loss of viability (Table S1). Again, this supports the view that the calcineurin pathway acts to prevent over activation of chitin synthesis and loss of viability. Growth inhibition due to caspofungin was increased in the *cna1*Δ and *crz1*Δ mutants, indicating that an intact Ca^2+^-calcineurin pathway was required for the ability of *C. albicans* to survive in the presence of caspofungin (Fig. 8C). An active calcineurin pathway therefore protects against caspofungin, reducing Mkc1 phosphorylation and limiting the production of supra-high chitin cells and the deposition of chitin in the outer cell wall (Fig. S1).

## DISCUSSION

The fungal cell wall is a remarkable organelle whose robustness provides physical protection, and the ability to penetrate and ramify in solid surfaces such as human tissues (Dantas et al., 2017). Yet, despite its rigid mechanical strength, it must remain sufficiently compliant and elastic to enable the surface to expand at sites of polarized growth and to accommodate sudden changes in external osmotic pressure by enabling expansion and contraction. These apparently antagonistic properties of rigidity and compliant elasticity require the architecture of the wall to be carefully constructed and regulated in response to environmental stresses (Ene et al., 2015). Here, we demonstrate a new paradigm in cell wall regulation in fungi suggesting that *C. albicans*, and by inference other fungi, have a mechanism to prevent the non-elastic carbohydrate polymer chitin to accumulate to levels in the wall that reduce fitness (Fig. S1B). The elasticity of the wall may also be affected by further induced changes in wall architecture such as polymer cross-linking and the regulation of chitin crystallinity.

Previously, we contributed to the discovery that *C. albicans* responds to echinocandin treatment by upregulating the content of chitin in the wall (Walker et al., 2010, 2015; Lee et al., 2012; Fernandes et al., 2014; Munro et al., 2007; Plaine et al., 2008). As a consequence of damage to β-1,3-glucan synthesis, cells compensate for the potential loss of wall integrity by upregulating chitin synthesis. However, we show here that cells with the very highest content of chitin progressively lose viability, as measured by PI staining, and cells with very high chitin content are not able to expand and generate high chitin buds. They can, however, retain some capacity to generate buds of normal chitin content when caspofungin stress is removed. The production of excessive chitin therefore correlates with the loss of fitness. But, under some conditions that stimulate the upregulation of chitin synthesis (e.g. treatment with CaCl_2_+CFW), this loss of fitness is prevented by a mechanism that attenuates the formation of supra-high chitin levels. We showed that the mechanism that attenuates chitin synthesis requires the Ca^2+^-calcineurin pathway, which dampens chitin upregulation by negatively regulating the CWI pathway.

Caspofungin treatment leads to rapid phosphorylation of Mkc1 and activation of the CWI pathway in *C. albicans* to upregulate genes associated with cell wall biogenesis and repair (Bruno et al., 2006; Liu et al., 2005), including CHS genes (Munro et al., 2007; Walker et al., 2008). However, unpublished results from our lab showed that overexpression of single *CHS* genes does not lead to increased chitin content, suggesting that transcriptional upregulation of chitin synthesis may require a stoichiometric increase of multiple proteins in the chitin synthase complex. Furthermore, this increase in CHS gene expression is accompanied by increases in Chs activity, cell wall chitin content (Walker et al., 2008) and cell wall remodeling that leads to increased exposure of chitin in the outer layers of the cell wall (Mora-Montes et al., 2011).

The increase in cell wall chitin seen in caspofungin-treated cells is accompanied by a decrease in β-1,3-glucan (Fig. S4), which may impact the overall elasticity of the wall. Cells with a two- to fourfold increase in chitin content had increased tolerance to caspofungin, but a proportion of cells that had higher chitin content became positive for PI and were unable to grow in the presence of the drug, demonstrating a correlation between excessively high chitin and the loss of fitness (Fig. S1B). However, cells with high chitin could generate buds or filaments of normal chitin content after wall stressors (CaCl_2_+CFW or caspofungin) were removed. Therefore, cells normally maintain levels of chitin in the cell wall below a threshold level that would negatively impact growth and division of the cells.

Upregulation of chitin synthesis by activation of the Ca^2+^-calcineurin pathway with CaCl_2_+CFW pre-treatment resulted in alterations in the amount and location of chitin in the wall and protected cells against subsequent caspofungin treatment (Figs 2 and 8). Some of the chitin formed in response to caspofungin exhibited significantly enhanced staining with the high molecular weight lectin WGA, indicating that increased amounts of chitin was accessible at the wall surface, and no longer restricted in its distribution in the inner cell wall. Chitin is a rigid linear polysaccharide that is hydrogen bonded in anti-parallel chains and is cross-linked to β-1,3 glucan (Gow et al., 2017). We speculate that, when chitin is distributed throughout the wall, this rigid lattice inhibits cell expansion with consequences to cell fitness. It has been suggested previously that chitin has a major effect on cell wall rigidity, and may account for the more flexible nature of the cell wall of buds compared with mother cells (Touhami et al., 2003; Chaudhari et al., 2012). But it is also possible that rigidity may be influenced by other cell wall properties that are regulated under these conditions, such as chitin-glucan cross-linking.

The expression of chitin synthase genes is regulated by multiple quasi-redundant signaling pathways (Munro et al., 2007). Although the calcineurin pathway can activate CHS gene expression (Munro et al., 2007), we show that it can also prevent supra-high chitin formation occurring – most likely by negatively regulating the CWI pathway, which is the primary positive regulator of chitin synthesis (Fig. 3). These results suggest that the Ca^2+^-calcineurin pathway regulates chitin content and functions as an attenuator, limiting the overproduction of chitin that would compromise cell viability. We show that the mechanism that inhibits overexpression of chitin also inhibits the CWI pathway and *CHS2-* and *CHS3*-dependent chitin synthesis (Figs 6 and 7).

The *cna1*Δ, *cnb1*Δ and *crz1*Δ mutants, which had moderately elevated chitin content, displayed higher tolerance to caspofungin (Fig. S1A), suggesting that tolerance could occur *in vivo* if a combination therapy of calcineurin inhibitors and caspofungin was used (Steinbach et al., 2007; Fortwendel et al., 2009; Juvvadi et al., 2019). As reported by others, we observed that an intact Ca^2+^-calcineurin pathway was required for full caspofungin tolerance (Sanglard et al., 2003; LaFayette et al., 2010), supporting the use of these inhibitors to avoid the ‘paradoxical effect’ associated with caspofungin treatment above the MIC. In support of the observation that overexpression of chitin synthesis is deleterious, we showed that deletion of *CHS3* or *MKC1* genes resulted in increased caspofungin tolerance of FK506-treated cells. These observations are in accordance with other reports showing that cross-talk between these two pathways might drive maintenance of viability in drug resistant *C. albicans* (LaFayette et al., 2010; Kumar et al., 2014). However, it is recognized that calcium ions act as a second messenger that can affect numerous aspects of cellular physiology, and have the potential to modulate physiology in ways that do not require calcineurin. It is also possible that cell wall stress is detected directly in the wall by an as yet unknown sensor that activated chitin synthesis in a calcineurin-independent manner (Fig. S1).

We therefore propose a model where the level of chitin in the cell wall is normally maintained within boundaries by the net effect of both positive and negative regulation of chitin synthesis (Fig. S1B). This regulatory equilibrium allows chitin to be upregulated under conditions of cell wall stress to levels that mitigate the effects of cell wall damaging agents and events (e.g. exposure to environmental secondary metabolites and antifungals, cell wall-degrading enzymes and changes in osmotic pressure). But it also maintains chitin levels in the wall below a threshold that would negatively affect the viability of *C. albicans* cells. The Ca^2+^-calcineurin pathway apparently plays a central role in this mechanism: positively activating CHS gene expression and negatively regulating the CWI pathway.

## MATERIALS AND METHODS

### Strains, media and growth conditions

*C. albicans* strains used in this study are listed in Table S2. Strains were maintained on YPD agar [1% (w/v) yeast extract, 2% (w/v) mycological peptone, 2% (w/v) glucose, 2% (w/v) agar]. Conditional mutant strains of the GRACE library were grown in liquid YPD supplemented with 1 µg/ml doxycycline for 24 h and subcultured on YPD agar supplemented with 1 µg/ml doxycycline for a further 24 h to downregulate *tet* promoter activity (Roemer et al., 2003). Doxycycline supplementation was then used in subsequent experiments to maintain suppression of the expression of key target genes. For chitin induction experiments, exponential phase cells were treated with CaCl_2_+CFW [0.2 M CaCl_2_ and 100 µg/ml Calcofluor White (fluorescent brightener 28, Sigma-Aldrich)] in liquid YPD for 12 h at 30°C in an incubator shaking at 200 rpm to increase their chitin content (Munro et al., 2007).

### Construction of the *chs3*Δ*0* mutant

A heterozygous *CHS3*/*chs3*Δ0 mutant was constructed using a PCR based method adapted from Noble and Johnson (2005). Primers MDL22 and MDL23 (Table S3) with 100 bp homology to the sequence immediately upstream of the start codon and 100 bp immediately downstream of the stop codon of *CHS3* were designed to anneal to sequences immediately adjacent to the *Candida dubliniensis HIS1* marker in pSN52 (Noble and Johnson, 2005). The resulting PCR product was transformed into *C. albicans* strain BWP17 (Wilson et al., 1999). His^+^ colonies were screened by PCR using primers MDL32 and MDL29 to confirm that one copy of *CHS3* had been replaced by the *CdHIS1* marker (Table S3). The resulting strain was designated *CHS3*/*chs3*Δ*0* (NGY490).

The second *CHS3* was disrupted using the mini ura-blaster method (Wilson et al., 2000). The disruption cassette containing *dpl200-URA3-dpl200* flanked by 100 bp of sequence homologous to the region of *CHS3* immediately 5′ of the start codon and 100 bp of sequence homologous to the region of *CHS3* immediately 3′ of the stop codon was PCR amplified from pDDB57 (Wilson et al., 2000) using primers MDL252 and MDL253 (Table S3). The PCR product was transformed into the *CHS3*/*chs3*Δ*0* heterozygous mutant. Ura^+^ colonies were screened by PCR using primers MDL32 and MDL183 (Table S3).

### Construction of *cch1*Δ/*mid1*Δ/*fig1*Δ/CIp10 mutants

*FIG1* was disrupted by amplification of the *dp1200 URA3* mini-blaster cassette from plasmid pDDB57 using primers with 19 bp homology to the cassette and 111 bp homology to *CaFIG1* (described by Brand et al., 2007). Two rounds of transformation in the *cch1*Δ/*mid1*Δ Ura− background (Brand et al., 2007), were followed by re-integration of *URA3* on the StuI-linearized CIp10 plasmid at the *RPS1* locus to give strain A243 (Brand et al., 2004).

### Construction of *yvc1*Δ/*yvc1*Δ/CIp10 mutant

The *YVC1* ORF and flanking regions were amplified from *C. albicans* SC5314 genomic DNA by primer pairs YVC-XHO/YVC-XBA and cloned into pBluescript KS+ to obtain pDS988. The ORF was deleted by inverse PCR with pDS988 as template using the primer pairs YVC-BG/YVC-PST. The 3.7 kb PstI–BglII fragment containing the URA3-blaster cassette from pMB7 (Coste et al., 2004) was cloned in the PCR product digested with the same enzymes to obtain plasmid pDS989. The plasmid was linearized with ApaI and SacI to liberate the deletion cassette that was transformed into *C. albicans* CAF4-2. The *URA3* marker was recycled by 5-FOA selection on FOA agar, generating DSY2655, and a second round of deletion generated the homozygous *YVC1* mutant, DSY2656. Further removal of the *URA3* marker generated the Ura^−^ isolate DSY3891. *URA3* was re-integrated on the CIp10-URA3 plasmid into the *RPS1* locus to generate strain A344.

### Microfluidics imaging

Yeast cells were grown to exponential phase in YPD medium with shaking at 200 rpm, washed in media and counted in a hemocytometer. Y04C microfluidic plates (Millipore Merck) were connected to an Onix microfluidic perfusion system (CellASIC, USA) and wells washed with medium (with or without cell stressors) by sealing the plate and applying 2×6 psi for 10 s, followed by 2 min at 5 psi for each well. Samples of 5×10^4^ *C. albicans* cells (100 μl) were loaded into the cell well by applying a 6 psi pulse for 10-15 s in the microfluidic plate. Media were perfused at 2 psi during treatment with cell wall stressors and/or recovery media. Samples were visualized using a 60× objective (×600 total magnification) by DIC field and by fluorescence using a DAPI and PI filter set, respectively (Chroma Technology), in a DeltaVision Core microscope (Applied Precision). Images were taken using CoolSNAP camera (Photometrics) and analyzed using DeltaVision software (SoftWorx version 5.0.0). The exposure time used in fluorescent pictures was kept constant throughout the videos to enable relative chitin content to be compared between images (DAPI filter set: 5%, 0.005 ms for CaCl_2_+CFW treatment; 5%, 0.5 ms for untreated and 3.2 µg/ml caspofungin-treated cells).

### Cell wall composition

Determination of cell wall mannan, chitin and β-glucan content was achieved by acid-hydrolyzing the polymers, and quantifying mannose, glucosamine and glucose content, respectively, by high-performance anion-exchange liquid chromatography with pulsed amperometric detection (HPLC)z as previously described (Mora-Montes et al., 2011).

### Fluorescence microscopy

*C. albicans* cells were fixed with 10% (v/v) neutral buffered formalin solution (Sigma-Aldrich) and exposed to 100 µg/ml FITC-conjugated wheat germ agglutinin (WGA, Sigma-Aldrich) for 60 min to stain chitin that was exposed at the cell surface to 25 µg/ml CFW for 5 min to stain total cell wall chitin. CFW is a small, permeable fluorochrome that binds chitin, whereas WGA is a high molecular weight chitin-binding lectin that does not penetrate into the deep wall and can therefore be used as a probe for exposed cell chitin (Mora-Montes et al., 2011). Vectashield (Vector Laboratories) mounting medium for fluorescence was added to minimize photobleaching. Samples were visualized by DIC field and by fluorescence using standard FITC and DAPI filter set, respectively (Chroma Technology), in a DeltaVision Core microscope (Applied Precision). Images were taken using CoolSNAP camera (Photometrics) and analyzed by DeltaVision software (SoftWorx version 5.0.0). The exposure time used in fluorescent pictures was kept constant for all samples to enable relative chitin content to be compared.

### Measurement of relative cell wall chitin content by FACS

Cell wall chitin content was measured by FACS analysis using a BD LSRII cytometer (BD Biosciences). Untreated, CaCl_2_+CFW-treated and caspofungin-treated cells were harvested by centrifugation, washed twice, resuspended in 1 ml FACS buffer (1× PBS; 0.5 mM EDTA; 0.5% BSA; 0.01% Tween 20) and stored at 4°C (Kaloriti et al., 2012). Immediately before injection in the LSRII cytometer, samples were sonicated (sonic dismembrator, Artek Systems) to break up cell clumps, and 10 µl of each sonicated sample was added to separate tubes containing 500 µl FACS buffer, 12.5 µg of CFW and 0.25 µg of propidium iodide (PI, Sigma-Aldrich) as a vital stain for 3-5 min.

Heat-killed *C. albicans* cells (100°C for 30 min) were stained with PI and used as a reference control for PI-stained non-viable/dead cells. Live untreated *C. albicans* cells stained with CFW were used as a reference control for CFW-stained cells. Samples containing 10 µl of unstained *C. albicans* cells plus 500 µl FACS buffer were also used as unstained controls. The PerCP-A detector was used to detect PI staining and the Indo-1-violet A detector for CFW staining. A total of at least 50,000 cells per sample were analyzed by FACS according to the BD LSRII manufacturer's guidelines (BD Biosciences).

Results were analyzed using BD FACS Diva software, according to BD Biosciences' guidelines, followed by analysis using FlowJo software (v10.1r7 64bit). In FlowJo, cells were first gated according to their size (FSC versus SSC) and second by cell viability (PerCP-A^−/+^). Relative chitin content (median fluorescence intensity – MFI) of live (PerCP-A^−^) or dead (PerCP-A^+^) cells is represented in graphs as the ratio between CaCl_2_+CFW or caspofungin-treated and untreated cells.

Viability of CaCl_2_+CFW- or caspofungin-treated cells was represented as the ratio between live unstained (PerCP-A^−^) cells and total *Candida albicans* cells analyzed (PerCP-A^−/+^) for each of the treatments.

### Determination of *C. albicans* growth in the presence of CaCl_2_+CFW or caspofungin

Caspofungin was serially diluted in water (0.015-16 µg/ml) in 96-well flat bottomed microtiter plates. *C. albicans* cultures that were treated or untreated with CaCl_2_+CFW were diluted and inoculated in 2×YPD to a final concentration of 1×10^5^ cells/ml, and 100 µl samples of these cultures were added to 96-well plates containing water and/or caspofungin. Plates were incubated at 30°C for 24 h, and optical densities were read in a microplate reader (Versa max, Molecular Devices) at 600 nm. Percentage growth was represented by the ratio of OD_600_ values for each pairing of cell populations, as stated in figure legends, where OD_600_ of untreated controls correspond to 100% of growth (value=1).

### Mkc1 phosphorylation

Protein extracts were prepared as described previously (Munro et al., 2007), and phosphorylated Mkc1 was detected by western blotting using an anti-phospho-p44/42 MAPK (Erk1/2, Thr202/Tyr204) antibody (1:1000; Cell Signaling Technology). The blots were stripped and Hog1 was used as a loading control by probing with an anti-Hog1 antibody (1:1000; y-215, Santa Cruz Biotechnology).

### Statistical analysis

All data were analyzed with GraphPad Prism statistical software (GraphPad Software). Increase in chitin content was compared against the control using one-way ANOVA test with Dunnett's post-hoc *t*-test. Significance levels were determined as *P*≤0.05.

## Supplementary Material

Supplementary information

Reviewer comments
