# Supplementary material for: Crosstalk between the calcineurin and cell wall integrity pathways prevents chitin overexpression in Candida albicans
Source: J Cell Sci. 2021 Dec 16;134(24):jcs258889. doi: 10.1242/jcs.258889 (PMC8729787; doi:10.1242/jcs.258889)
Supplement: Supplementary information [file joces-134-258889-s1.pdf]

A

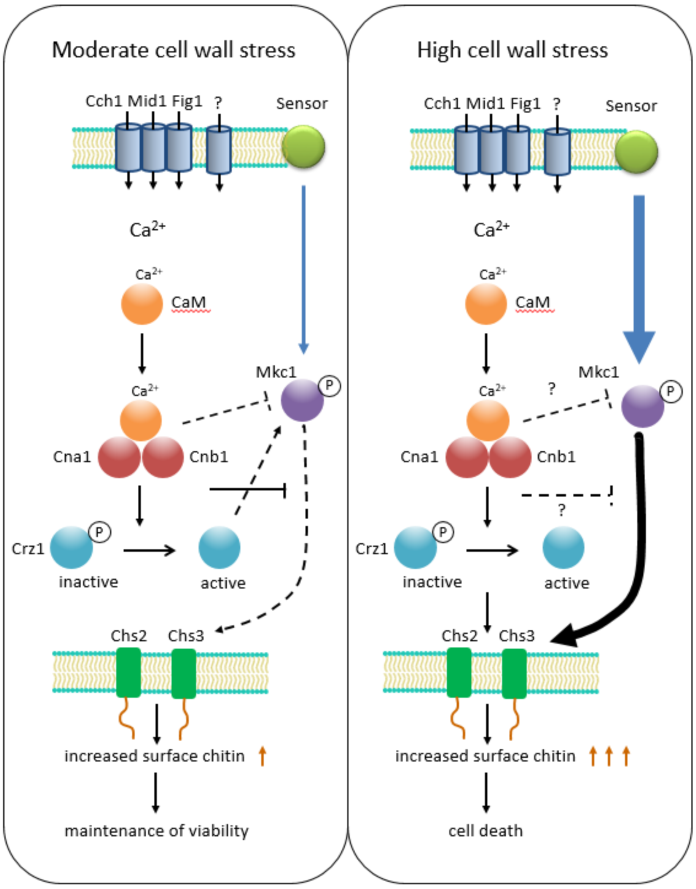

B

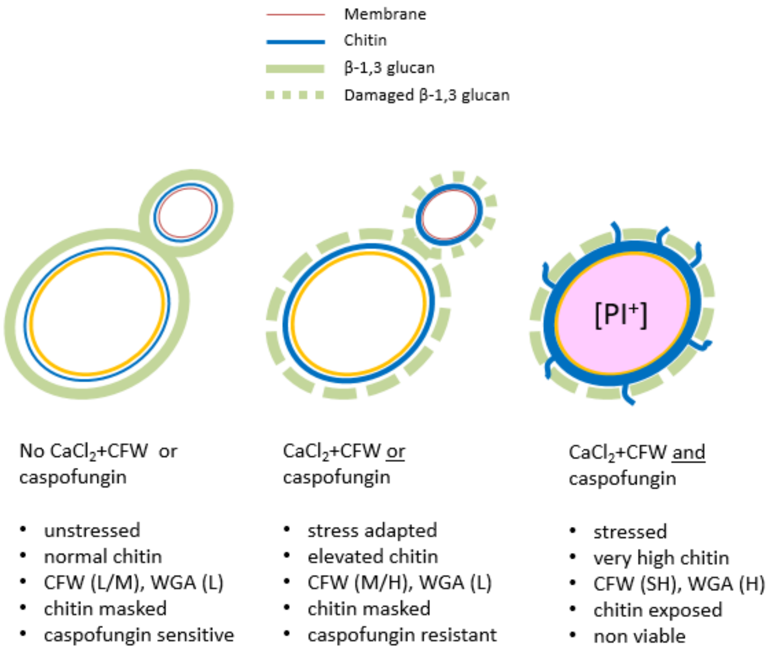

Journal of Cell Science • Supplementary information

Fig. S1. Model for attenuation of chitin synthesis and relationship with cell viability. A. Activation of Ca^{2+}-calcineurin and cell wall integrity

pathways activates chitin synthase gene expression, resulting in increased chitin levels in the cell wall and in its outer layers, making it accessible to WGA (left). Activation of chitin synthesis via the cell wall integrity pathway may also occur via an unknown sensor in the cell wall under conditions of high cell wall stress. Synthesis of chitin results in cells with supra-high chitin cell wall levels, which are non-viable (right). The Ca2+-calcineurin pathway attenuates chitin synthesis preventing the formation of supra-high chitin cells, by negatively regulating the cell wall integrity pathway (left). This down regulates the synthesis of chitin which is then located only in the inner layers of the cell wall. B. Model based on data presented describing the relationship between chitin accumulation, sensitivity to caspofungin and cell viability. Intensity of

CFW staining of cells classified as low (L), medium (M), high (H) or supra-high (SH). WGA staining is normally inhibited by β-1,3 glucan but occurs when chitin becomes exposed at the surface.

Journal of Cell Science • Supplementary information

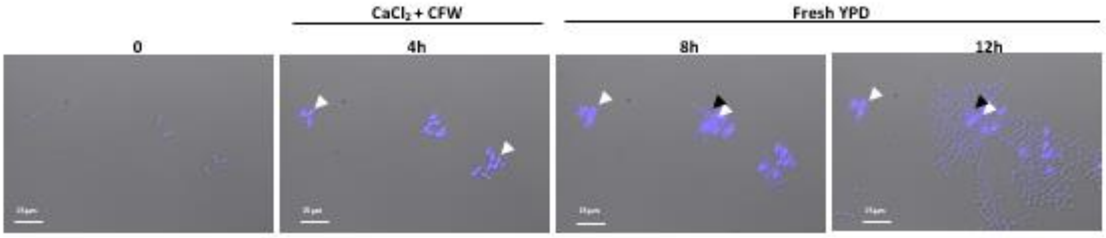

Fig. S2. Supra-high chitin cells do not resume growth even in the absence of cell wall stress. Representative micrographs at indicated time points from microfluidics experiments where *C. albicans* wild-type cells were treated with 0.2 M $CaCl_2$ + 100 µg/ml CFW for 6 h, followed by growth in YPD for 6 h in the absence of $CaCl_2$+CFW. Images are shown at 4 h, 8 h and 12 h. Total chitin of *C. albicans* was visualised using CFW staining. White arrows indicate growth arrested or dead supra-high chitin cells and black arrows indicate low chitin daughter cells formed when stress is removed.

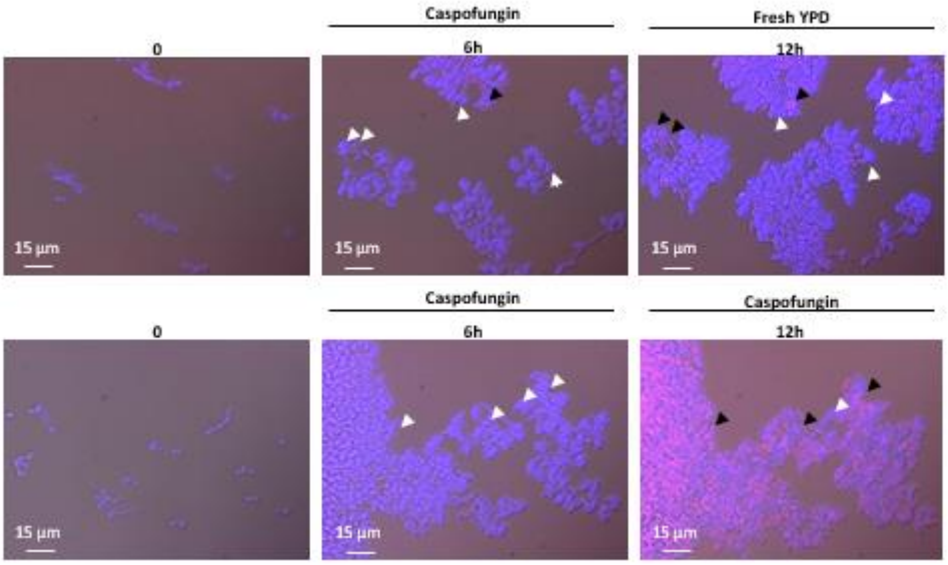

Fig. S3. Caspofungin treatment results in PI positive cells that do not resume growth. Representative micrographs at indicated time points from microfluidics experiments where *C. albicans* wild-type cells were treated with 3.2 µg/ml of caspofungin for 6 h, followed by removal of treatment and growth on YPD for 6 h, or caspofungin for 12 h. Images are shown at 6 h and at 12 h. Total chitin of *C. albicans* was visualised using CFW staining and loss of viability using PI. White arrows indicate high chitin cells that are viable and black arrows indicate dead supra-high chitin cells.

Journal of Cell Science • Supplementary information

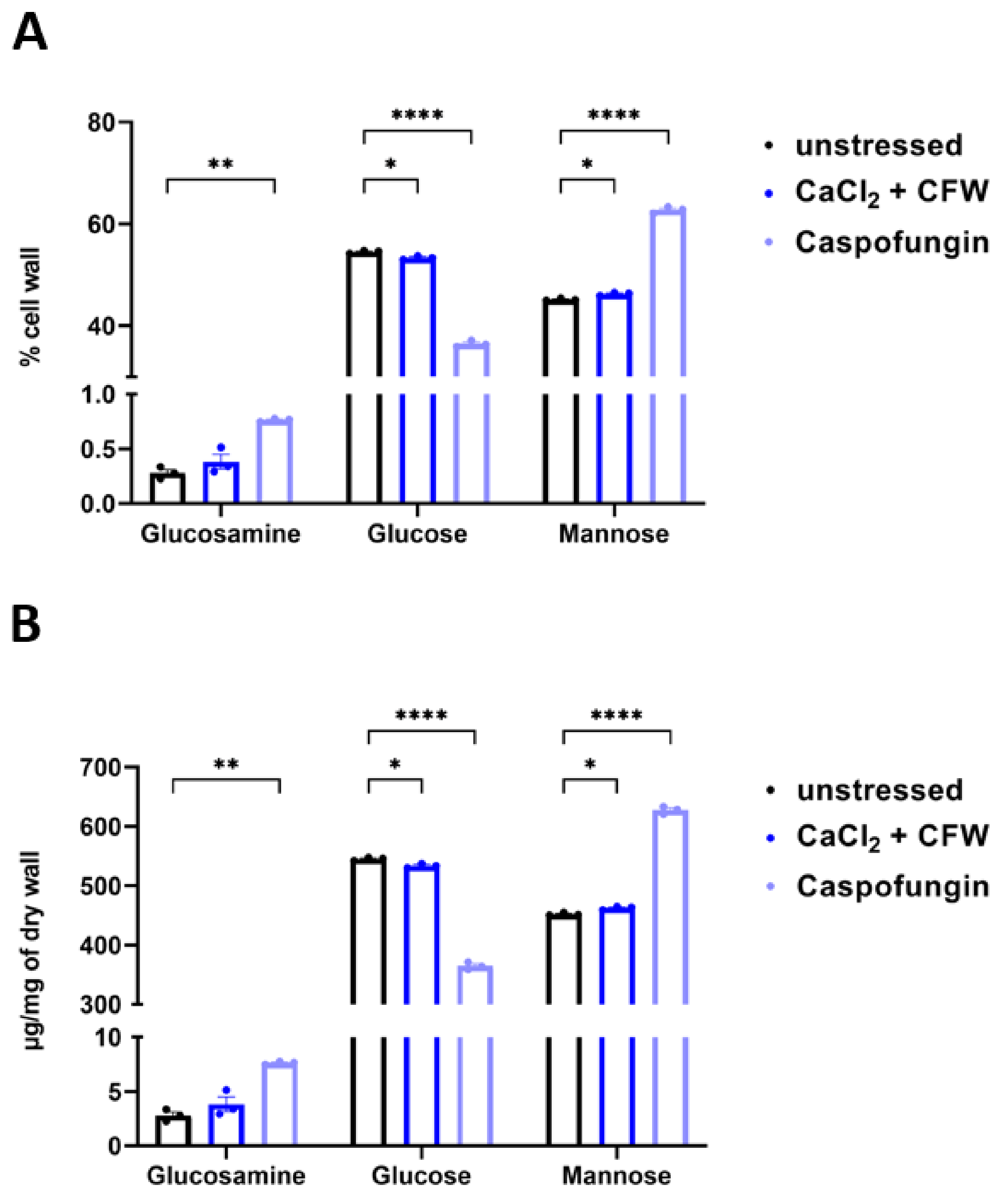

Fig. S4. Cell wall composition of *C. albicans* cells grown in the presence of cell wall stressors. *C. albicans* cells were grown in the presence of 0.2 M $CaCl_2$ + 100 µg/ml CFW or caspofungin (2 µg/ml). Cell walls were hydrolysed and the monosaccharide contents measured by HPLC expressed as (A) percentage of total cell wall sugars and (B) micrograms per 1 mg dry weight. Data represent mean of results from at least 3 replicates (*p ≤ 0.05, **p ≤ 0.01, ****p ≤ 0.001).

Table S1. Changes in chitin content and loss of viability in mutants of the calcineurin pathway after treatment of yeast cells with 0.2 M CaCl2 and 100 µg/mL CFW for 12 h. Values are SEMs (n=4 independent experiments). Significance compared to the wild-type strain * p≤0.05, ** p≤0.01

Strain	Loss off viability on Ca +CFW treatment (%)	Fold-change in chitin content (live cells)	Fold-change in chitin content (dead cells)
wt	3.9 ± 2.7	2.20 ± 0.20	21.32 ± 4.46
cna1Δ	33.18 ± 5.79**	9.74 ± 5.53*	14.36 ± 6.11
cnb1Δ	26.88 ± 11.84**	10.37 ± 4.13*	19.28 ± 13.58
crz1Δ	25.9 ± 8.27**	11.44 ± 5.02*	12.65 ± 3.57

Table S2. *C. albicans* strains used in this study.

Strain	Strain name	Genotype	Source
**Controls**			
Wild-type	SC5314	URA3/URA3	Fonzi and Irwin, 1993
CAI4/Clp10	NGY152	*ura3Δ::imm434/ura3Δ::imm434 iro1/iro1Δ::imm434*	Brand *et al*, 2004
BWP17		*ura3Δ::imm434/ura3Δ::imm434, his1::hisG/his1::hisG, arg4::hisG/arg4::hisG*	Wilson *et al.*, 1999
**Signalling mutants**			
cek2Δ		*cek2Δ/cek2Δ*	Yi *et al.*, 2008
cek1Δ	CK43B-16	*ura3/ura3 cek1Δ::hisG-URA3-hisG/cek1Δ::hisG*	Csank *et al.*,1998
hog1Δ	JC45	*ura3Δ::imm434/ura3Δ::imm434, his1::hisG/his1::hisG, hog1Δ::loxP-URA3-loxP/hog1Δ::loxP-HIS1-loxP*	Enjalbert *et al.*,2006
mkc1Δ	CM1613	*mkc1Δ::hisG-CaURA3-hisG/mkc1Δ::hisG ura3Δ::imm434/ura3Δ::imm434*	Navarro-Garcia *et al.*,1995
cph1Δ	JKC19	*cph1Δ /cph1Δ*	Liu *et al.*,1994
sko1Δ	VIC100	*ura3Δ::imm434/ura3Δ::imm434 his1Δ::hisG/his1 Δ::hisG sko1Δ::hisG/sko1Δ::hisG–URA3–hisG*	Alonso-Monge *et al.*,2010

Journal of Cell Science • Supplementary information

rck2Δ	BH4 (CA6)	*rck2::hisG/rck2::hisG*	Li *et al.*, 2008
msn4Δ	MS17 (MSC8)	*ura3::imm434/ura3::imm434 ade2::hisG/ade2::hisG/msn4 Δ::hisG/msn4 Δ::hisG*	Nicholls *et al.*, 2004
swi6Δ	Het.	*swi6Δ::HIS3/SAT-1-TETp-SWI6*	Xu *et al.*, 2007
swi4 Δ	Het.	*swi4Δ::HIS3/SAT-1-TETp-SWI4*	Xu *et al.*, 2007
cch1Δ	NGY166	*cch1Δ ::hisG/cch1Δ::hisG/ cch1Δ::hisG, rps1Δ::CIp10/RPS1*	Brand *et al.*, 2007
mid1Δ	NGY167	*mid1Δ::hisG/mid1Δ::hisG, rps1Δ::pCIp10/RPS1*	Brand *et al.*, 2007
fig1Δ	NGY372	*fig1Δ::dp1200/fig1Δ::dp1200, rps1 Δ::CIp10/RPS1*	Brand *et al.*, 2007
pmr1Δ	NGY355	*pmr1Δ::hisG/pmr1Δ::hisG, RPS1/rps1Δ::CIp10-URA3*	Bates *et al.*, 2005
yvc1Δ	A3441	*yvc1Δ::hisG/yvc1Δ::hisG, RPS1/rps1Δ::CIp10-URA3*	This study
cch1Δ mid1Δ fig1Δ	A243	*cch1Δ::hisG/cch1Δ::hisG/ cch1Δ::hisG/mid1Δ::hisG/mid1Δ::hisG fig1Δ::hisG/ fig1Δ::hisG rps1Δ::pCIp10/RPS1*	This study
rcn1Δ	JLR37.1		Reedy *et al.*, 2010
cna1Δ	MKY379	*cna1Δ::hisG/cna1Δ::hisG RPS1/rps1Δ::URA3*	Sanglard *et al.*, 2003
cnb1Δ	JRB64	*ura3Δ::imm434/ura3Δ::imm434 his1::hisG::HIS1/his1::hisG arg4::hisG/arg4::hisG cnb1Δ::UAU1/cnb1Δ::ARG4*	Cruz *et al.*, 2002
crz1Δ	MKY381	*crz1Δ::hisG/crz1Δ::hisG/RPS1/rps1Δ::URA3, crz1Δ::CRZ1-SAT1*	Karababa *et al.*, 2008
ras1Δ	Can52	*ras1-2/ras1-4 ura3Δ::λimm434/ura3Δ::λimm434 ras1Δ::hisG/ras1Δ::hph*	Feng *et al.*, 1999
tpk1Δ	IIHH6-4a	*tpk1Δ::hisG/tpk1Δ::hisG*	Bockmuhl *et al.*, 2001
tpk2Δ	AS1	*tpk2Δ::hisG/tpk2Δ::hisG*	Sonneborn *et al.*, 2000
efg1Δ	HLC52	*efg1Δ::hisG/efg1Δ::hisG*	Lo *et al.*, 1998
**Chitin synthase mutants**			

Journal of Cell Science • Supplementary information

chs2Δ	NGY7	*chs2Δ::hisG/chs2 Δ::hisG:: URA3::hisG*	Gow *et al.*, 1994
CHS3/chs3Δ0	NGY490	*CHS3/chs3Δ0::CdHIS1*	This study
chs3Δ	MLC16	*chs3Δ0::CdHIS1/chs3Δ0::dpl200-URA3-dpl200*	This study
chs8Δ	NGY125	*chs8Δ::hisG-URA3-hisG/chs8Δ::hisG*	Munro *et al.*, 2003
chs2Δ chs3Δ	C156	*chs2Δ::hisG/chs2Δ::hisG, chs3Δ::hisG/chs3Δ::hisG-URA3-hisG*	Mio *et al.*, 1996
chs2Δ chs8Δ	NGY137	*chs2Δ::hisG/chs2Δ::hisG*, *chs8Δ::hisG-URA3-hisG/chs8Δ::hisG*	Munro *et al.*, 2003

Journal of Cell Science • Supplementary information

Table S3. Primers used in this study as described in the Materials and Methods.

Primer name	Sequence (5'-3')
MDL22	TCC CTT GCA TTA TAC CAA AAC TTA TAG ACA GAC GAC AGA CAA CAG ACA ACC CGC TTA ATC TTC TTC TTC TAC ATT TAT TCC ATA TTC AAT ATC ATT CAC T GCT CGG ATC CAC TAG TAA CG
MDL23	CAA AAA TAT AAT CTG TCT TTT TTA TAT TGT TAA TAA TTT TAT ATA ACC ATA TAC ATA AAT AAA AGT CCT TTC TCT CTC TCT TTT TTA AGC TTT AAC CCA A CCA GTG TGA TGG ATA TCT GC
MDL32	CTATGTTGATTATACTATAGAAACTTAC
MDL29	CCATTTGGCTCTTCACTACC
MDL252	TCC CTT GCA TTA TAC CAA AAC TTA TAG ACA GAC GAC AGA CAA CAG ACA ACC CGC TTA ATC TTC TTC TTC TAC ATT TAT TCC ATA TTC AAT ATC ATT CAC T TTT CCC AGT CAC GAC GTT
MDL253	CAA AAA TAT AAT CTG TCT TTT TTA TAT TGT TAA TAA TTT TAT ATA ACC ATA TAC ATA AAT AAA AGT CCT TTC TCT CTC TCT TTT TTA AGC TTT AAC CCA A TGT GGA ATT GTG AGC GGA TA
MDL183	CCCAGTGAC ACCATGAGC
YVC-XHO	TAG AGA ACT CGA GTG TTA CAC GCA TCT CGA CGC
YVC-XBA	CTT TCT AGA CAC TGC CCA TTC CTG TAT TCG CC
YVC-BG	AGT AGA TCT TTC AAG CAA TGC CTG AACT
YVC-PST	AAA AAC TGC AGC TCT AAG CTT TAT TTT CAA

Journal of Cell Science • Supplementary information

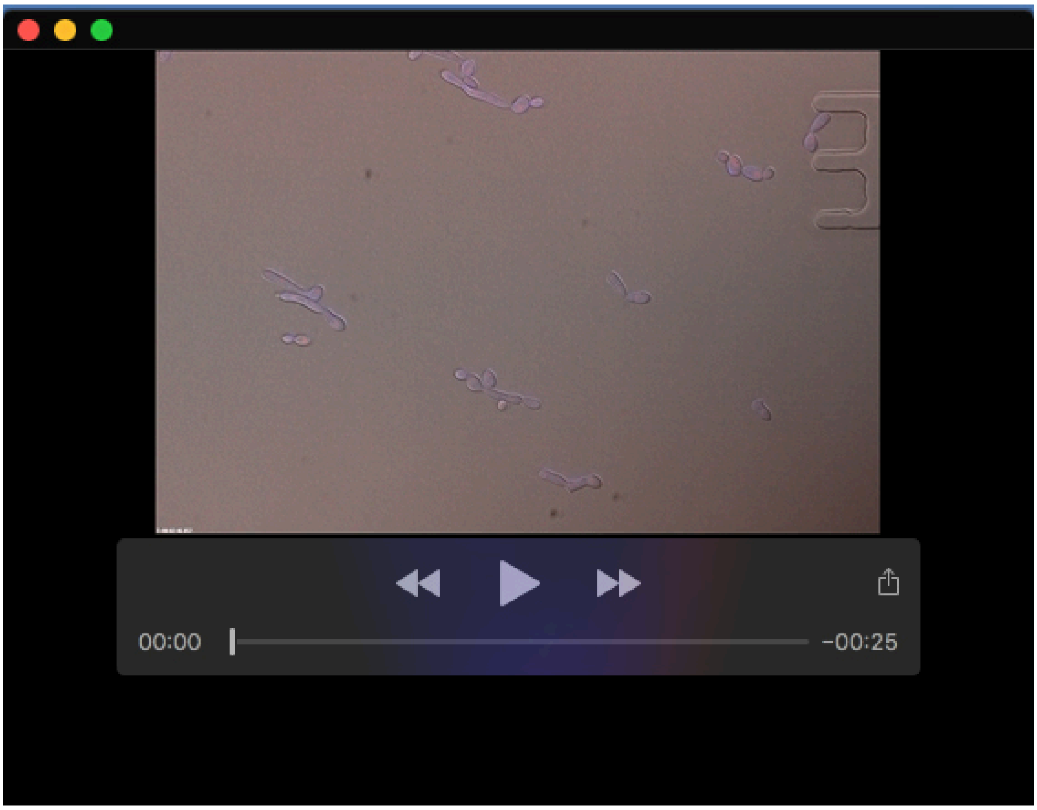

Movie 1. Caspofungin treatment of *C. albicans* cells for 6 h. Microfluidics experiments where *C. albicans* wild-type cells were grown in the presence of YPD for 2 h, treated with 3.2 µg/ml of caspofungin for 6 h, followed by removal of treatment and growth on YPD for 6 h. Total chitin of *C. albicans* was visualised by using CFW staining and loss of viability by using PI.

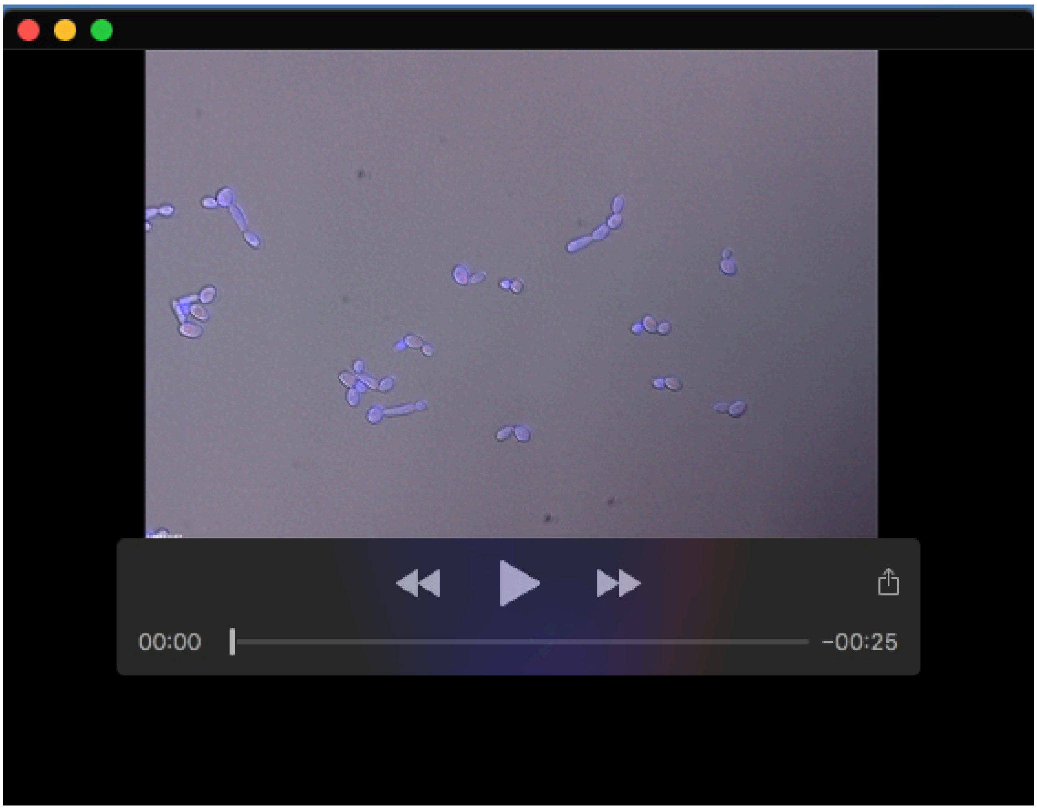

Movie 2. Caspofungin treatment of *C. albicans* cells for 12 h. Microfluidics experiments where *C. albicans* wild-type cells were grown in the presence of YPD for 2 h, treated with 3.2 µg/ml of caspofungin for 12 h. Total chitin of C. albicans was visualised by using CFW staining and loss of viability by using PI.

Journal of Cell Science • Supplementary information
