# Supplementary material for: Crosstalk between the calcineurin and cell wall integrity pathways prevents chitin overexpression in Candida albicans
Source: J Cell Sci. 2021 Dec 16;134(24):jcs258889. doi: 10.1242/jcs.258889 (PMC8729787; doi:10.1242/jcs.258889)
Supplement: Reviewer comments [file joces-134-258889_review_history.pdf]

Journal of Cell Science

Crosstalk between calcineurin and the cell wall integrity pathways prevents chitin overexpression in *Candida albicans*

Alessandra da Silva Dantas, Filomena Nogueira, Keunsook K. Lee, Louise A. Walker, Matt Edmondson, Alexandra C. Brand, Megan D. Lenardon and Neil A. R. Gow

DOI: 10.1242/jcs.258889

Editor: John Heath

Review timeline

Original submission:	7 May 2021
Editorial decision:	2 June 2021
First revision received:	24 August 2021
Editorial decision:	20 September 2021
Second revision received:	7 November 2021
Accepted:	9 November 2021

Original submission

First decision letter

MS ID#: JOCES/2021/258889

MS TITLE: Cross-talk between calcineurin and the cell wall integrity pathways prevents chitin overexpression and loss of fungal viability

AUTHORS: Alessandra da Silva Dantas, Filomena Nogueira, Keunsook Lee, Louise Walker, Matt Edmondson, Alexandra Christine Brand, Megan D Lenardon, and Neil Andrew Robert Gow
ARTICLE TYPE: Research Article

We have now reached a decision on the above manuscript.

To see the reviewers' reports and a copy of this decision letter, please go to: https://submit-jcs.biologists.org and click on the 'Manuscripts with Decisions' queue in the Author Area. (Corresponding author only has access to reviews.)

As you will see, the reviewers raise a number of substantial criticisms that prevent me from accepting the paper at this stage. They suggest, however, that a revised version might prove acceptable, if you can address their concerns. If you think that you can deal satisfactorily with the criticisms on revision, I would be pleased to see a revised manuscript. We would then return it to the reviewers.

We are aware that you may be experiencing disruption to the normal running of your lab that makes experimental revisions challenging. If it would be helpful, we encourage you to contact us to discuss your revision in greater detail. Please send us a point-by-point response indicating where you are able to address concerns raised (either experimentally or by changes to the text) and where you will not be able to do so within the normal timeframe of a revision. We will then provide further guidance. Please also note that we are happy to extend revision timeframes as necessary.

Please ensure that you clearly highlight all changes made in the revised manuscript. Please avoid using 'Tracked changes' in Word files as these are lost in PDF conversion.

© 2021. Published by The Company of Biologists under the terms of the Creative Commons Attribution License (https://creativecommons.org/licenses/by/4.0/).

I should be grateful if you would also provide a point-by-point response detailing how you have dealt with the points raised by the reviewers in the 'Response to Reviewers' box. Please attend to all of the reviewers' comments. If you do not agree with any of their criticisms or suggestions please explain clearly why this is so.

Reviewer 1

Advance summary and potential significance to field

It is a very interesting story on the effect of caspofungin on Candida. It uses for the first time a microfluidic system to decipher cell by cell the role of pathways. This is very smart because it allows to follow an heterogenous cell population with differential sensitivity to drugs under different medium changes. This paper is difficult to "digest" but easily enlighted by the supplementary Fig.1.However the role of many of the genes and pathways discussed were known: for example the effects of mutations in the Ca+2-calcineurin on chitin content or the interaction between the calcineurin and Mkc1 cell integrity pathways in controlling chitin production have been previously reported.

Comments for the author

Answers to most comments listed below will require an extensive experimental work .

1. Do the authors suggest that caspofungin treated cells are dying of an "indigestion" of high concentrations of chitin irrespectively of the modification of the concentration of beta 1,3 glucans by caspofungin? Are mutants of candida overexpressing chitin synthases more sensitive to caspofungin?
2. The reduction of growth without Ca/CFW pretreatment is not so big. In Fig.1, the amount of PI stained yeasts is quite high even after removal from the Ca/CFW condition. Are we sure that all the PI-labeled cells are dead? In Fig.1D a lot of cells are apparently PI-positive. It would have been nice to see the PI staining after 12h.
3. In fig.1 if you incubate longer the cells with the respective drugs, do you get all the cells with high amount of chitin dying? It is also strange to see that the number of cells that become PI-positive continue to increase when they were replaced in a fresh culture medium and it is different when the cells are incubated in CaCl2/CFW where the number of dead cells does not increase after they are put back in the fresh culture medium. Any suggestion for the existence of this sensitive subpopulation: younger or older cells? Cells with a different permeability? What happen if you leave the cells longer in the drug of Ca/CFW treatment?
4. Even though previous data by the Gow's lab justifies the joint use of Ca and CFW having a preincubation with Ca and CFW makes things more complicate to understand or dissect. For example what would happen if Ca or CFW were used alone?
5. Nikkomycin is reducing the amount of cell wall chitin because of the inhibition of all chitin synthases.
In presence of Nikkomycin, then the amount of chitin in the cell wall is reduced and based on the authors' theory such reduction in the level of chitin should be good for the fungus to respond to caspofungin treatment. But in fact the opposite happens since many publications have shown that there is a combinatorial effect of Nikkomycin and Caspofungin. Any explanation?!
6. It is strange that no localization or quantification of the beta 1,3 glucans is shown or even investigated in this experimental set up since caspofungin is a beta 1,3 glucan synthase inhibitor.
7. The authors have published in the past different images of chitin as short rods or long fibrils. Can some of the data obtained here indicated that different types of chitin are produced with a different localization of the three major forms of fibrils as seen in recent ssNMR publications. Do all these different chitin molecules stain with the same intensity with CFW?
8. The differences of growth of the different chs mutants are not so big. Did the authors look at the expression of the different chs genes under the conditions tested by the authors during the incubation with the drugs and then after removal of the drugs.
9. In fig 2B, could the difference of labeling with WGA be only due to differences in permeability of the cell wall induced by caspofungin?
10. Did the authors look at the morphology of the septa under the experimental conditions?
11. What is the role of swi6 here? Any connection with the Calcineurin pathway since the swi6 mutant and the calcineurin mutants have a similar phenotype in response to Ca/CFW? It would have

© 2021. Published by The Company of Biologists under the terms of the Creative Commons Attribution License (https://creativecommons.org/licenses/by/4.0/).

been nice to test another general cell cycle regulator such as Cas5 since Cas5 regulates changes in gene expression associated to cell wall stress through both Swi4/Swi6-dependent mechanisms

Reviewer 2

Advance summary and potential significance to field

The manuscript by Alessandra da Silva Dantas and colleagues from the Gow lab asks how the chitin content of the cell wall is upregulated and maintained in response to cell wall stress. The major result is that the calcineurin pathway, which was previously shown to contribute to upregulation of chitin synthesis in response to cell wall stress, also moderates this upregulation. In addition, calcineurin regulates chitin levels through the cell wall integrity pathway. Evidence presented also shows that calcium channels and upstream regulators of the calcineurin pathway do not modulate or attenuate cellular chitin levels. The experiments underlying these findings are generally solid and present convincing evidence of many of the conclusions that are drawn. However, there are several major questions that remain unaddressed, and some interpretations of the results need to be clarified in order for this to be considered the type of advance appropriate for JCS.

Comments for the author

Major issues:
1. The assumption that chitin accumulation is the cause of cell death in vitro has not been established. A clear correlation showing that strains which exhibit increased chitin content have reduced growth rate and vice versa, akin to the in vivo fitness experiment from the referenced Ben-Ami et al. 2011, would provide a stronger basis for the hypothesis that supra-high chitin levels are either tightly associated with and/or causative of death, rather than that they are simply a symptom of a higher degree of stress in caspofungin than in calcofluor white.
2. Figure 1 is summarized in lines 288-291 as follows: “These observations suggest that CaCl2+CFW treatment stimulates the appearance of viable high chitin cells, while high chitin levels seen in caspofungin treated cells was harmful to cells, causing growth arrest, loss of viability and ultimately cell death.”
However, figure 1A is difficult to interpret, due to the density of cells, and figure 1C indicates that in both conditions, high chitin cells are inviable. The source of cells for Fig. 1B is not clear – ideally, the the flow cytometric analysis for both stresses should be provided. As currently presented, a more balanced interpretation is that both treatments cause some cells to produce high levels of chitin (yet, intriguingly, far more death is observed with caspofungin exposure), and in both cases these high-chitin cells die.
3. All results compare between the responses to two different sources of stress at static concentrations. This raises the question of whether the chitin response is due to the level of stress or due to the nature of the chemicals as an alternative hypothesis, that the differences are simply due to the concentrations of caspofungin and CFW+CaCl2, was not mentioned. This distinction can be easily tested and would add much clarity to the subsequent conclusions.
4. The summarizing model (Figure S1) suggests that calcium uptake is important for chitin attenuation; yet, in figures 4 and 5 the mutants lacking either individual (or all three) calcium channels behave like the wild type. Thus these results do not support this aspect of the model. Please include a justification of why this point is included in the model and add the data to support it, or modify the model to fit the data. In either case, a discussion of other potential mechanisms for the pathway to sense chitin overproduction would help.
5. The title suggests that the findings here are relevant to all fungi, whereas the work was only done in C. albicans.

Additional issues:
1. The manuscript suffers from many errors in English usage as well as typographical errors. It would benefit from careful editing by the native English speaking authors.
2. Considering the central nature of the calcineurin pathway in this work, a brief overview of the pathway in the introduction, and what is known about its connection to the CWI pathway, would help put the work in context.
3. The presentation is sometimes confusing. Some examples:
a. Lines 459-460: chitin levels increase after treatment with CFW+CaCl2

© 2021. Published by The Company of Biologists under the terms of the Creative Commons Attribution License (https://creativecommons.org/licenses/by/4.0/).

“even in the presence of FK506” . Given that FK506 is a calcineurin inhibitor and calcineurin attenuates supra-high chitin production, this result is confirmatory.
Suggested instead: “Again we observed that total chitin levels and exposed chitin levels increased in CaCl2+CFW treated WT cells with or without FK506 (Fig. 6A).”
b. Lines 469-471: “Deletion of MKC1 partially mitigated growth inhibition associ-ated with CaCl2+CFW treatments, when grown in the presence of the calcineu-
rin inhibitor (+FK506) (Fig. 6B).” The text ignores the much stronger result: that the mkc1Δ strain growth is similar in the presence or absence of FK506, which is genetic evidence supporting that the CWI pathway is central to the calcineurin-mediated stress-responsive effect on viability. Suggest reorganizing fig 6B to highlight this result highlighting this important result.
5. Figure 8B shows that pre-treatment with CFW+CaCl2 reduces Mkc1 phosphorylation, even in calcineurin mutants, indicating that calcineurin is not the sole regulator of Mkc1 and the CWI pathway in response to CFW+CaCl2. This needs to be stated clearly and shown in any model.

Minor points:
1) There is an errant “0h” directly in the middle of figure 1A.
2) Figure 3A has 19 bars, but only 18 labels.
3) “mid1” in figure 4A and B is not italicized 4) The labels on the right side of figure 6A and 7A are incorrect and confusing (+Ca+2CFW), and also are inconsistent with other labels (Ca+2 instead of CaCl2)
5) Line 502 suggests that figure 2B shows a cna1 mutant, it does not.
6) Figure 8C is never referenced in the text.

First revision

<u>Author response to reviewers' comments</u>

<u>Response to reviewers comments: Da Silva et al</u>
We thank the journal review panel for the helpful and useful comments. Acting on those has improved the clarity of the manuscript. It is also evident and appreciated from the referee’s comments that they were also aware of previous work in our lab that establishes some of the background to this study. We have fully revised the test and undertaken more microfluidic and cell wall analysis experiments to address the questions that were raised.

Reviewer 1

General comments. Advance Summary and Potential Significance to Field: It is a very interesting story on the effect of caspofungin on Candida. It uses for the first time a microfluidic system to decipher cell by cell the role of pathways. This is very smart because it allows to follow a heterogeneous cell population with differential sensitivity to drugs under different medium changes. This paper is difficult to “digest” but easily Enlighted by the supplementary Fig.1. However the role of many of the genes and pathways discussed were known: for example the effects of mutations in the Ca^{+2}-calcineurin on chitin content or the interaction between the calcineurin and Mkc1 cell integrity pathways in controlling chitin production have been previously reported.

Thank you for the endorsement of our overall approach. We have made several textual changes to further clarify the narrative and made more cross references to Supplementary Figure 1 which the referee felt was helpful in digesting the complexities of the multiple pathways involved in this response (lines 127, 238, 284, 320, 372, 400, 471). We would be happy to include this Figure within the main manuscript if requested to do so. Of course, the pathways under study have been the subject of many studies from a number of labs, including our own, however what is novel and new in our report is how the calcineurin and PKC pathways interact with each other to control chitin overexpression. The importance of our study is therefore in the new finding calcineurin pathway is a negative regulator of chitin synthesis and this occurs via inhibition of the Mkc1 pathway.

© 2021. Published by The Company of Biologists under the terms of the Creative Commons Attribution License (https://creativecommons.org/licenses/by/4.0/).

Comments for the Author.

We thank the referee for insights and suggestions. We have made a wide range of corrections, re-analysed some data sets and performed more experiments including some with longer term incubations and wall analyse to measure chitin and ß-1,3 glucan content.

1. Do the authors suggest that caspofungin treated cells are dying of an "indigestion" of high concentrations of chitin irrespectively of the modification of the concentration of beta 1,3 glucans by caspofungin?

We have argued that chitin and ß-1,3 glucan are cantilevered [Lenardon et al 2021] and that because chitin is a non-elastic antiparallel-folded polysaccharide that a complete girdle of chitin would be deleterious to the cell, by restraining cell expansion. In contract to chitin, ß-1,3 glucan has a degree of elasticity due to its tri-helical structure. Several papers have shown that ß-1,3 glucan levels are lower following caspofungin treatment, however, we have undertaken a further HPLC analysis of the walls of treated and control cells under our experimental conditions to examine the ratio of ß-1,3 glucan and chitin (Supplementary Fig. 4) and these confirm that there is a significantly increased amount of chitin relative to ß-1,3 glucan, suggesting the wall would be more rigid. This shows that chitin/glucan levels of $CaCl_2$+CFW treated cells are 0.716% compared to caspofungin treated cells (2.094%) vs untreated cells (0.514%). We have added a note in the text to make this clearer (lines 150- 158, 172-181, 229-231, 442-443, 446-450). We have undertaken further time lapse microfluidics experiments that very high content yeast cells do not regrow, but can survive and release normal chitin buds when $CaCl_2$+CFW and caspofungin are removed (see below). This further underlines that a rigid girdle of chitin may prevent growth.

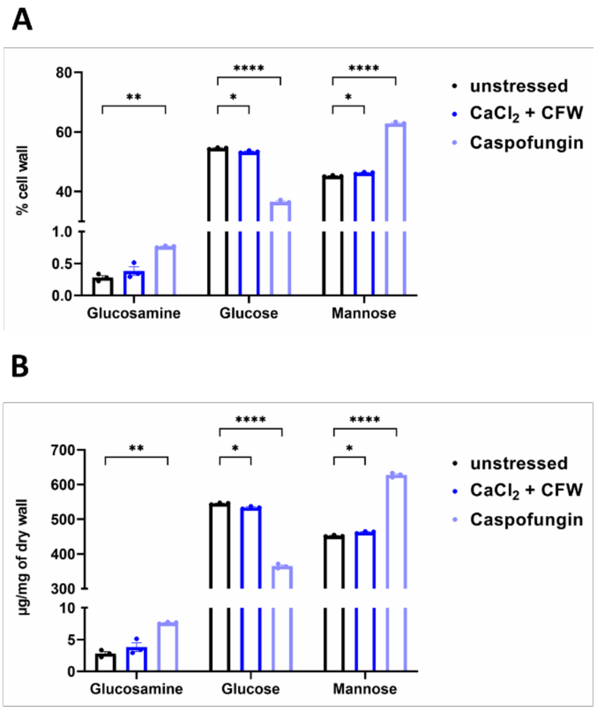

Supplementary Figure 4. Cell wall composition of *C. albicans* cells grown in the presence of cell wall stressors. *C. albicans* cells were grown in the presence of 0.2 M $CaCl_2$ + 100 µg/ml CFW or caspofungin (2 µg/ml). Cell walls were hydrolysed and the monosaccharide contents measured by HPLC expressed (A) percentage of total cell wall sugars and (B) micrograms per 1 mg dry weight. Data represent mean of results from at least 3 replicates (*$p < 0.05$, **$p < 0.01$, ****$p < 0.001$).

Are mutants of candida overexpressing chitin synthases more sensitive to caspofungin?

We have shown previously that *CHS* promoters are activated by stimuli that induce the calcineurin pathway and PKC pathway [Munro et al, 2027; Lenardon et al, 2009]. But unpublished results from our lab showed that overexpression of chitin synthase *CHS3* using the *GAL1* inducible promoter had

© 2021. Published by The Company of Biologists under the terms of the Creative Commons Attribution License (https://creativecommons.org/licenses/by/4.0/).

no effect on chitin content. Ono et al (2000) showed that overexpression of *CHS4* (which is not a structural chitin synthase gene) increased chitin content. Therefore, transcriptional upregulation of chitin synthesis may require a stoichiometric increase of multiple proteins in the chitin synthase complex. Upregulation of CHS genes do not lead to an increased chitin content. We, and others, have shown previously that other methods of increasing chitin synthesis by altering physiological growth conditions can increase chitin content and reduces caspofungin sensitivity [Walker et al, 2008 and here). We have added a note in the Discussion to underline these points (lines 434-437).

2. The reduction of growth without Ca/CFW pre-treatment is not so big. In Fig.1, the amount of PI stained yeasts is quite high even after removal from the Ca/CFW condition. Are we sure that all the PI-labeled cells are dead? In Fig.1D a lot of cells are apparently PI-positive. It would have been nice to see the PI staining after 12h.

The reviewer is correct that we interpret PI positivity as an indicator of reduced viability or death. Again, we have shown previously that most PI positive cells permanently lose viability (Walker et al., 2008) although we do not contest that a small fraction of cells may be able to recover. But, we see a strong correlation between becoming PI positive and the inability to resume budding. The corollary is also the case – PI negative cells can resume budding in the presence of wall stressors. We note also that caspofungin (and CaCl2+CFW) causes cell cycle arrest and that apparently delays some of cells becoming PI positive. When growth resumed cells became chitin-rich and increasingly PI positive. To reinforce this we have now monitored PI staining over 12h using microfluidics (Movies 1-2, line 142-146, 150-153). This shows that some cells become PI positive when treated with Caspofungin for 6h and remain so after flushing with growth medium lacking caspofungin media for another 6h. Furthermore when cells were treated with caspofungin for 12h we could observe increasing numbers of PI positive cells. [Supplementary Figure 3].

In these new experiments we recorded no occasions where PI positive cells started to grow again. Also, high chitin cells did not resume growth when caspofungin was flushed out (data not shown). But, daughters of normal levels of chitin could be formed from the high chitin cells. We also undertook experiments in which chitin was stimulated with CaCl2+CFW. After flushing out of CaCl2+CFW cells did reinitiate growth, but, interestingly formed filaments, even at 30°C. We interpret this filamentation response to the release of a general cell stress response. But, as with caspofungin treatment, individual cells with very high chitin levels again did not grow.

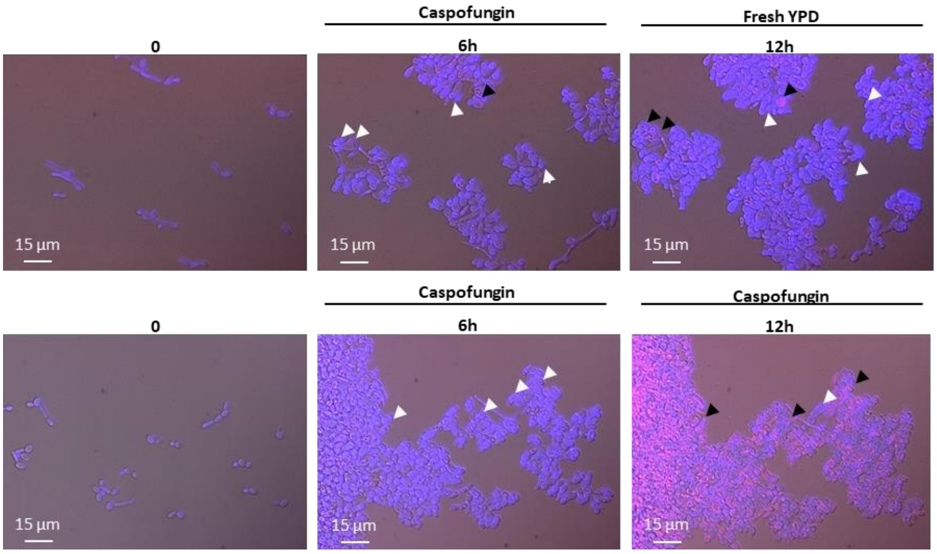

Supplementary Figure 3. Caspofungin treatment results in PI positive cells that do not resume growth. Representative micrographs of microfluidics experiments where *C. albicans* wild-type cells were treated with 3.2 µg/ml of caspofungin for 6h, followed by removal of treatment and growth on YPD for 6h, or caspofungin for 12h. Total chitin of *C. albicans* was visualised by using CFW staining and loss of viability by using PI. White arrows indicate high chitin cells that are viable and black arrows indicate dead supra-high chitin cells.

© 2021. Published by The Company of Biologists under the terms of the Creative Commons Attribution License (https://creativecommons.org/licenses/by/4.0/).

3. In fig.1 if you incubate longer the cells with the respective drugs, do you get all the cells with high amount of chitin dying?

We see progressive increase in chitin upregulation and PI positivity (see above). This was again in the new experiments that were performed. This is a stochastic phenomenon and we do not see or expect all cells to die. We have endeavoured to ensure we do not give that impression in the manuscript (lines 142-148, 150-158).

It is also strange to see that the number of cells that become PI-positive continue to increase when they were replaced in a fresh culture medium and it is different when the cells are incubated in CaCl2/CFW where the number of dead cells does not increase after they are put back in the fresh culture medium. Any suggestion for the existence of this sensitive subpopulation: younger or older cells? Cells with a different permeability? What happen if you leave the cells longer in the drug of Ca/CFW treatment?

See discussion above. Cell cycle arrested cells don't lose viability as quickly since apparently you have to grow to die under the experimental conditions being examined. This would be analogous to observations made with other cell wall acting antimicrobials including penicillin where growth inhibitors compromise the efficacy of the ß-lactams. Death (PI positivity) is the consequence of attempted growth when the cell has a high chitin cell wall. When we add Caspofungin and CFW simultaneously the cells die they die more quickly.

Cells with a different permeability? This is an interesting comment. We do not know if the cells have altered permeability, but will consider how this might be addressed in the future.

4. Even though previous data by the Gow's lab justifies the joint use of Ca and CFW having a preincubation with Ca and CFW makes things more complicate to understand or dissect. For example, what would happen if Ca or CFW were used alone?

We showed previously (Munro et al 2007), that Ca^{2+}alone activates transcription of *CHS1*, *CHS2*, *CHS3* and *CHS8* and that the calcineurin specific inhibitors FK506 and Cyclosporin A inhibited this transcription. We also showed that transcription of *CHS2* and *CHS3* was activated in part via the Ca^{2+}-calcineurin/Crz1 pathway and that Crz1p repressed the expression of *CHS1*. In addition, transcription from all *CHS* promoters was reduced in the *cna1*Δ mutant background in response to exogenous Ca^{2+}. In this publication, we also tested the effects of combinations of $CaCl_2$ and CFW treatments and showed that all four promoters were hyperstimulated by combined treatment with $CaCl_2$ and CFW (Munro et al 2007, Table 3) and combinations of $CaCl_2$ and CFW resulted in a threefold increase in the amount of chitin in the cell wall (Munro et al 2007, Figure 7). We also found in the current study that CFW pre- treatment can protect against caspofungin killing, but not as efficiently as with $CaCl_2$ and CFW combinations (not shown). The general conclusion is that combinations of $CaCl_2$ and CFW act synergistically in all measureable phenotypes, and we refer also to published information for supporting evidence for this.

5. Nikkomycin is reducing the amount of cell wall chitin because of the inhibition of all chitin synthases. In presence of Nikkomycin, then the amount of chitin in the cell wall is reduced and based on the authors' theory such reduction in the level of chitin should be good for the fungus to respond to caspofungin treatment. But in fact the opposite happens since many publications have shown that there is a combinatorial effect of Nikkomycin and Caspofungin. Any explanation?!

The referee is correct, and we have also reported this result. The answer to the question reflects a proposed sweet spot for the levels of chitin in the cell. Under cell wall stress conditions due to caspofungin exposure, an increase of approximately two fold in the wall chitin is clearly protective (e.g. Walker et al 2008; Walker et al., 2013). But, we show here, that for supra-high chitin – beyond that level of chitin, there is a negative effect on cell viability. We see synergistic negative effects on viability between CFW and caspofungin – for same reason.

6. It is strange that no localization or quantification of the beta 1,3 glucans is shown or even investigated in this experimental set up since caspofungin is a beta 1,3 glucan synthase inhibitor.

© 2021. Published by The Company of Biologists under the terms of the Creative Commons Attribution License (https://creativecommons.org/licenses/by/4.0/).

The absolute level of ß-1,3 glucan in supra high chitin content cells may not be relevant. If the wall becomes non-elastic because of a greatly elevated chitin content then the ß-1,3 glucan may have little effect on cell viability. None the less we have now measured the chitin/ ß-1,3 glucan ratio by HPLC of cell wall extract in supra-high chitin cells (see above) [Supplementary Figure 4).

7. The authors have published in the past different images of chitin as short rods or long fibrils. Can some of the data obtained here indicated that different types of chitin are produced with a different localization of the three major forms of fibrils as seen in recent ssNMR publications. Do all these different chitin molecules stain with the same intensity with CFW?

This is not easy to answer with precision. Chitinase treatment of chitin fibrils leads to an increase in Calcofluor White (CFW) binding, not a decrease. This is because chitinase treatment may initially create more frayed ends of chitin, by loosening the tightly hydrogen bonded antiparallel chin microfibrils resulting in an increased elevated CFW binding potential. So multiple aspects of chitin architecture have the potential to affect the relative biding of CFW. However, to our knowledge, and based on our previous studies, there is no significant difference in the intensity of CFW binding of the short and long forms of chitin microfibrils (Lenardon et al 2007).

8. The differences of growth of the different chs mutants are not so big. Did the authors look at the expression of the different chs genes under the conditions tested by the authors during the incubation with the drugs and then after removal of the drugs.

As above there is only a weak relationship between transcriptional level, chitin levels and growth rate (see Munro et al., 2007), implying that translational regulation is especially important in wall regulation. This seems intuitively reasonable since cell wall damage must lead to rapid activation of protective responses to prevent irreversible loss of viability. This whole area is interesting, but we do not feel this would alter our interpretations of what we have set out in the manuscript, but it may inspire future studies.

9. In fig 2B, could the difference of labeling with WGA be only due to differences in permeability of the cell wall induced by caspofungin?

As mentioned above, we do not know what the effects on wall permeability are. From our previous work (e.g. Walker et al 2018; Yadav et al., 2020) it seems like the main contribution to cell wall permeability resides in the properties and architecture of the outer cell wall mannoprotein later, rather than the inner chitin- ß-1,3 glucan cell skeletal layer.

10. Did the authors look at the morphology of the septa under the experimental conditions?

Thank you for this thought. We did not originally look at this. But, we have now scrutinised many images and microfluidic movies of septa that were used to generate the data sets presented in the paper and we find no obvious differences in the location, distribution or architecture of septa.

11. What is the role of swi6 here? Any connection with the Calcineurin pathway since the swi6 mutant and the calcineurin mutants have a similar phenotype in response to Ca/CFW? It would have been nice to test another general cell cycle regulator such as Cas5 since Cas5 regulates changes in gene expression associated to cell wall stress through both Swi4/Swi6-dependent mechanisms.

The *swi6* mutant and the calcineurin mutants do have a similar phenotype in response to $CaCl_2$+CFW, (green bar in Fig. 3) but they do not accumulate chitin to very high levels. We interpreted that as reinforcing our assertion that the cell wall integrity pathway plays a role in the chitin attenuation mechanism. But, the referee is alluding to a potentially fertile area for an in-depth bespoke further investigation.

Reviewer 2

Advance Summary and Potential Significance to Field: The manuscript by Alessandra da Silva Dantas and colleagues from the Gow lab asks how the chitin content of the cell wall is upregulated and maintained in response to cell wall stress. The major result is that the calcineurin pathway, which

© 2021. Published by The Company of Biologists under the terms of the Creative Commons Attribution License (https://creativecommons.org/licenses/by/4.0/).

was previously shown to contribute to upregulation of chitin synthesis in response to cell wall stress, also moderates this upregulation. In addition, calcineurin regulates chitin levels through the cell wall integrity pathway. Evidence presented also shows that calcium channels and upstream regulators of the calcineurin pathway do not modulate or attenuate cellular chitin levels. The experiments underlying these findings are generally solid and present convincing evidence of many of the conclusions that are drawn. However, there are several major questions that remain unaddressed, and some interpretations of the results need to be clarified in order for this to be considered the type of advance appropriate for JCS.

Reviewer 2 Comments for the Author. Major issues:

1. The assumption that chitin accumulation is the cause of cell death *in vitro* has not been established. A clear correlation showing that strains which exhibit increased chitin content have reduced growth rate and vice versa, akin to the *in vivo* fitness experiment from the referenced Ben-Ami *et al*. 2011, would provide a stronger basis for the hypothesis that supra- high chitin levels are either tightly associated with and/or causative of death, rather than that they are simply a symptom of a higher degree of stress in caspofungin than in calcofluor white.

The referee challenges us to go beyond our study to speculate or demonstrate conditions that might mimic our lab conditions and drive this loss of viability due to greatly elevated chitin under physiological conditions. This is both interesting and worthwhile of further investigation. However, our assertion that super-high chitin leads to loss of viability is based on the strong correlation we demonstrate between having greatly elevated levels of chitin in the wall and the accumulation of PI positive cells. We have reinforced with the new data that are now presented, where we show progressive increase in PI positive cells in the presence of caspofungin and that the cells with the highest chitin are only able to grow when the echinocandin stress is removed and a normal chitin cell is formed (Supplementary Figure 2, lines 150-158, 446-450).

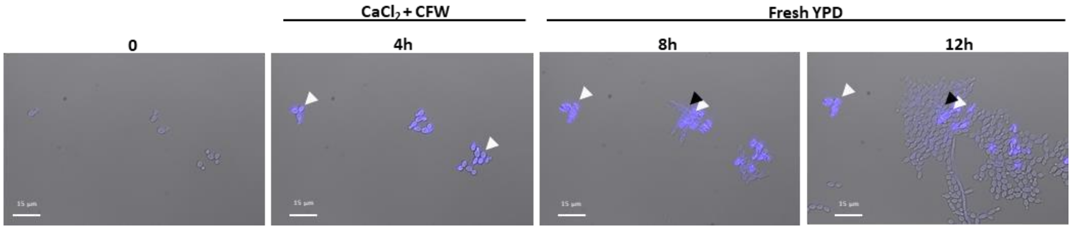

Supplementary Figure 2. Supra-high chitin cells do not resume growth even in the absence of cell wall stress. Representative micrographs of microfluidics experiments where *C. albicans* wild-type cells were treated with 0.2 M $CaCl_2$ + 100 µg/ml CFW for 6 h, followed by growth on YPD for 6 h in the absence of $CaCl_2$ + CFW. Total chitin of *C. albicans* was visualized by using CFW staining. White arrows indicate growth arrested or dead supra-high chitin cells and black arrows indicate low chtin daughter cells formed when stress is removed.

We underline that this loss of viability does not occur when only moderate levels of chitin elevated are induced – indeed we, and others, have shown that moderate chitin increase is protective against caspofungin, because they can compensate for inflicted damage on the chitin-glucan wall skeleton (Walker *et al*., 2008, Walker *et al*., 2013). We have not undertaken any *in vivo* experiments at this stage to simulate conditions representing a therapeutic intervention with an echinocandin - which we feel is appropriate for a discovery science project that aims to establish a new paradigm. We show that multiple stresses are required to achieve levels of chitin that compromises viability, and that the loss of viability is normally prevented by a previously unrecognised attenuation mechanism that normally establishes a ceiling for chitin content in the wall to prevent pathological levels of chitin from accumulating. It is not unreasonable to speculate that this mechanism may be widely spread or general in the fungi. We do not comment on the *in vivo* conditions that may or may not mimic the *in vitro* conditions we deploy. We only seek to demonstrate that the wall has a homeostatic mechanism that serves to maintain chitin concentration in the wall within physiological boundaries.

© 2021. Published by The Company of Biologists under the terms of the Creative Commons Attribution License (https://creativecommons.org/licenses/by/4.0/).

2. Figure 1 is summarized in lines 288-291 as follows: “These observations suggest that CaCl2+CFW treatment stimulates the appearance of viable high chitin cells, while high chitin levels seen in caspofungin treated cells was harmful to cells, causing growth arrest, loss of viability and ultimately cell death.” However, figure 1A is difficult to interpret, due to the density of cells, and figure 1C indicates that in both conditions, high chitin cells are inviable.

We see the ambiguity that is pointed out and have reworded lines 142-148, 150-158, 442-443, 483-485, 488-490 to clarify. This confusion arises due to the point (also made above), that moderate increases in chitin are not detrimental to the cell and indeed are partially protective against damage caused by caspofungin. We refer also to the new showing that high chitin cells become progressively nonviable from 6h- 12h – and don’t bud.

The source of cells for Fig. 1B is not clear – ideally, the flow cytometry analysis for both stresses should be provided. As currently presented, a more balanced interpretation is that both treatments cause some cells to produce high levels of chitin (yet, intriguingly, far more death is observed with caspofungin exposure), and in both cases these high-chitin cells die.

We have changed the legend to Fig. 1B (lines 665-666) to clarify the source of the cells. The referee is right that stimulation via multiple stressors is required to elevate chitin to a level that impacts cell viability. As mentioned above, this has been further underlined in several places in the revised manuscript. The source of Fig 1B has been added (lines 665-666). Both treatments cause cells to produce high levels of chitin, but as caspofungin also reduces ß-13 glucan levels, the ratio chitin/glucan is higher than for cells treated with CaCl2+CFW treated cells. These observations are supportive of the hypothesis that a more rigid wall eventually results in cell death.

3. All results compare between the responses to two different sources of stress at static concentrations. This raises the question of whether the chitin response is due to the level of stress or due to the nature of the chemicals as an alternative hypothesis, that the differences are simply due to the concentrations of caspofungin and CFW+CaCl2, was not mentioned. This distinction can be easily tested and would add much clarity to the subsequent conclusions.

We see the point. We have no doubt that we would see concentrations-dependent effects since we have demonstrated that the effects (for example) of CaCl2+CFW addition are synergistic rather than additive. Our hypothesis is that there is a threshold level of chitin in the wall that, when exceeded, starts to impact on viability. We again point to foundation publications summarised above (e.g. Munro *et al.*, 2007; Walker *et al.*, 2008). We feel that repeating some of these early experiments titrating the concentration of each of the stressors (Ca^{2+} and CFW and caspofungin) would not change the conceptual advance or conclusions of the study. We do not pretend that each stressor works differently and there are also aspects of the pathological phenotype that differs. For example, we have noted, but not pursued an observation in our microfluidic experiments using different concentrations of caspofungin where we see the concentration dependent induction of vacuolation. This was not seen with CaCl2+CFW treatments. Also, our new experiments show that release from stress imposed by CaCl2+CFW results in filament formation – but not so when cells are released from caspofungin stress. This will be followed up in future studies.

4. The summarizing model (Figure S1) suggests that calcium uptake is important for chitin attenuation; yet, in figures 4 and 5 the mutants lacking either individual (or all three) calcium channels behave like the wild type. Thus, these results do not support this aspect of the model. Please include a justification of why this point is included in the model and add the data to support it, or modify the model to fit the data. In either case, a discussion of other potential mechanisms for the pathway to sense chitin overproduction would help.

This is correct. We deduce that the response is mediated via the known calcium channels. There are other wall stress detectors in the wall, and potentially other possible calcium ion channels. Calcium can also be released from internal stores. We know that calcineurin is however required for the attenuation mechanism and that exogenous calcium does modulate chitin synthesis transcription (Munro *et al.*, 2007). We have modified the figure accordingly. We have also change the summarizing model (Supplementary Figure 1, lines 310-314).

© 2021. Published by The Company of Biologists under the terms of the Creative Commons Attribution License (https://creativecommons.org/licenses/by/4.0/).

5. The title suggests that the findings here are relevant to all fungi, whereas the work was only done in C. albicans.

We agree that we have not demonstrated the generality of the findings. The summary statement makes it clear that we use *Candida* as the model organism. We have changed the Abstract and title to underline this as well. Lines 1-2 and 39.

Additional issues:
1. The manuscript suffers from many errors in English usage as well as typographical errors. It would benefit from careful editing by the native English speaking authors.

The manuscript has been revised, line by line, by English speaking authors to further refine the text.

2. Considering the central nature of the calcineurin pathway in this work, a brief overview of the pathway in the introduction, and what is known about its connection to the CWI pathway, would help put the work in context.

Revised as requested (lines 99 -103, 949-951).

3. The presentation is sometimes confusing. Some examples: a.Lines 459-460: chitin levels increase after treatment with CFW+CaCl2 "even in the presence of FK506". Given that FK506 is a calcineurin inhibitor and calcineurin attenuates supra-high chitin production, this result is confirmatory. Suggested instead: "Again we observed that total chitin levels and exposed chitin levels increased in CaCl2+CFW treated WT cells with or without FK506 (Fig. 6A)."

Revised as suggested. Lines 331-332.

4. Lines 469-471: "Deletion of MKC1 partially mitigated growth inhibition associated with CaCl2+CFW treatments, when grown in the presence of the calcineurin inhibitor (+FK506) (Fig. 6B)." The text ignores the much stronger result: that the mkc1Δ strain growth is similar in the presence or absence of FK506, which is genetic evidence supporting that the CWI pathway is central to the calcineurin-mediated stress-responsive effect on viability. Suggest reorganizing fig 6B to highlight this result highlighting this important result.

Revised as suggested. Lines 340-343.

5. Figure 8B shows that pre-treatment with CFW+CaCl2 reduces Mkc1 phosphorylation, even in calcineurin mutants, indicating that calcineurin is not the sole regulator of Mkc1 and the CWI pathway in response to CFW+CaCl2. This needs to be stated clearly and shown in any model.

We have changed the text (lines 392-395) to state that other inputs and pathways could also regulate the Mkc1/CWI pathway.

Minor points:

1) There is an errant "0h" directly in the middle of figure 1A.

Corrected

2) Figure 3A has 19 bars, but only 18 labels.

Our apologies – now corrected.

3)"mid1" in figure 4A and B is not italicized

Corrected

4) The labels on the right side of figure 6A and 7A are incorrect and confusing (+Ca+2CFW), and

© 2021. Published by The Company of Biologists under the terms of the Creative Commons Attribution License (https://creativecommons.org/licenses/by/4.0/).

also are inconsistent with other labels (Ca+2 instead of CaCl2)

These have been redone and made consistent as suggested. Thank you for pointing this out.

5) Line 502 suggests that figure 2B shows a cna1 mutant, it does not.

Corrected to Fig 2B (wt), 8A (cna1 null) line 378

6) Figure 8C is never referenced in the text.

This is now mentioned in the revised text - line 398

Cited references:
Lenardon, M.D., Whitton, R.K.,Munro, C.A., Marshall, D. & Gow, N.A.R. (2007). Individual chitin synthase enzymes synthesise microfibrils of differing structure in the fungal cell wall. *Molecular Microbiology* **65**, 1164-1173.

Lenardon, M.D. Sood, P., Dorfmueller, H., Brown, A.P.J. & Gow, N.A.R. (2020). Scalar nanostructure of the *Candida albicans* cell wall; a molecular, cellular and ultrastructural analysis and interpretation. *The Cell Surface*. 6, 10047. https://doi.org/10.1016/j.tcsw.2020.100047

Munro, C.A., Selvaggini, S., de Bruijn, I., Walker, L., Lenardon, M.D., Gerssen, B, Milne, S., Brown A.P.J. & Gow, N.A.R. (2007). The PKC, HOG and Ca^{2+} signalling pathways coordinately regulate chitin synthesis in *Candida albicans*. *Molecular Microbiology* **63**, 1399- 1413.

Ono, N., et al (2000). The yeast Chs4 protein stimulates the trypsin-sensitive activity of chitin synthase 3 through an apparent protein-protein interaction. Microbiology 146: 385-391. doi: 10.1099/00221287-146-2-385.

Walker, L., Munro, C.A., Lenardon, M., de Bruijn, I., McKinnon A., & Gow, N.A.R. (2008). Stimulation of chitin synthesis rescues *Candida albicans* from echinocandins. *PLoS Pathogens* **4**(4): e1000040. doi:10.1371/journal.ppat.1000040.

Walker, L.A., Gow, N.A.R. & Munro, C.A. (2013). Elevated chitin content reduces the susceptibility of *Candida* species to caspofungin. *Antimicrobial Agents and Chemotherapy* **57**, 146-154.

Walker, L.A, Sood P., Lenardon, M.D., Milne, G., Olson. J., Jensen, G., Wolf, J, Casadevall, A, Adler-Moore, J. & Gow, N.A.R. (2018). The viscoelastic properties of the fungal cell wall allow traffic of AmBisome as intact liposome vesicles. *MBio*, **9** (1) e02383-17.

Yadav, B., Mora-Montes, H.M., Wagener, J., Cunningham, I., West, L., Haynes, K., Brown, A.J.P. &. Gow, N.R.R. (2020). Differences in fungal immune recognition by monocytes and macrophages: *N*-mannan can be a shield or activator of immune recognition. *The Cell Surface* **6**, 100042 https://doi.org/10.1016/j.tcsw.2020.100042

Second decision letter

MS ID#: JOCES/2021/258889

MS TITLE: Crosstalk between calcineurin and the cell wall integrity pathways prevents chitin overexpression in Candida albicans

AUTHORS: Alessandra da Silva Dantas, Filomena Nogueira, Keunsook Lee, Louise Walker, Matt Edmondson, Alexandra Christine Brand, Megan D Lenardon, and Neil Andrew Robert Gow
ARTICLE TYPE: Research Article

We have now reached a decision on the above manuscript.

© 2021. Published by The Company of Biologists under the terms of the Creative Commons Attribution License (https://creativecommons.org/licenses/by/4.0/).

To see the reviewers' reports and a copy of this decision letter, please go to: https://submit-jcs.biologists.org and click on the 'Manuscripts with Decisions' queue in the Author Area. (Corresponding author only has access to reviews.)

As you will see, both reviewers continue to raise a number of substantial criticisms that prevent me from accepting the paper at this stage. They both raise important issues of experimental design and interpretation that have not been addressed in the revised paper. These can be addressed by a thorough editorial revision of the paper to provide a more accurate interpretation of the data.

If you think that you can deal satisfactorily with the criticisms on revision, I would be pleased to see a revised manuscript. We would then return it to the reviewers. However, to save you time, you may wish to withdraw the paper at this stage as we cannot entertain further rounds of revision.

We are aware that you may be experiencing disruption to the normal running of your lab that makes experimental revisions challenging. If it would be helpful, we encourage you to contact us to discuss your revision in greater detail. Please send us a point-by-point response indicating where you are able to address concerns raised (either experimentally or by changes to the text) and where you will not be able to do so within the normal timeframe of a revision. We will then provide further guidance. Please also note that we are happy to extend revision timeframes as necessary.

Please ensure that you clearly highlight all changes made in the revised manuscript. Please avoid using 'Tracked changes' in Word files as these are lost in PDF conversion.

I should be grateful if you would also provide a point-by-point response detailing how you have dealt with the points raised by the reviewers in the 'Response to Reviewers' box. Please attend to all of the reviewers' comments. If you do not agree with any of their criticisms or suggestions please explain clearly why this is so.

Reviewer 1

Advance summary and potential significance to field

The use of a microfluidic system to better understand the effect of an anti fungal drug is smart with a kinetic approach is smart and this is an important development. The analysis of the role of chitin synthesis in fungal death should be pursued and that the Gow's lab will answer many of the questions asked in this review. The fact that this ms lead to many unsolved questions is indeed for me a "+" since it may stimulate interesting cell wall research in the future.

Comments for the author

This reviewer thanks the authors for improving the clarity of the ms which unfortunately remains very puzzling; it is a very difficult piece of work to read and understand and I am still not totally convinced by the conclusions presented.
The role of chitin here remains difficult to recognize: sometimes it is good for the fungus sometimes it is bad for growth and all this is only based on very little differences in the amount of chitin. In addition, the additional data giving a quantification of the amount of chitin in the cell wall remains imperfect since they are only relative: Ca+CFW gives a 0.7% increase in chitin / b1,3 glucan; caspo a 2.0% increase. But is it due to a reduction of b1,3 glucan consecutive to the caspo treatment rather than a true weight increase in chitin ie mg of chitin per mg of cell wall? The increase in chitin remains truly moderate and many fungi have a very high amount of chitin in their cell wall without being perturbed in their growth.
It even leads to a provocative comment. Are we sure that it is the chitin increase which is responsible for the growth inhibition or are we only looking at a secondary compensatory event due to an environmental stress which has nothing to do with the fungal death which could then be due to another cause?
The authors suggest that the increase in chitin induced a modification of the elasticity of the cell wall which would be responsible for the death of the fungus but this "cell wall elasticity" is not quantified.

© 2021. Published by The Company of Biologists under the terms of the Creative Commons Attribution License (https://creativecommons.org/licenses/by/4.0/).

Similarly, the authors speculate that the modification of the cell wall could be due to an alteration of the links between chitin and ß-1,3 glucan but this is purely hypothetical since no chemical data are supporting this statement.
The role of chitin synthase genes is not also clear especially when the authors write in their response to a reviewer that the upregulation of chitin synthase genes does not lead to an increase in chitin". Similarly the authors interpret a better WGA staining by an increase in chitin of the external layer of the cell wall. It may only be due to an increased permeability of the cell wall to WGA due to a modification of the permeability of the cell wall resulting from the deleterious effect of the antifungal drug. Could the UDP-GlcNAc synthesis pathway be stimulated by caspofungin and be the only one responsible for the chitin increase?
For the calcineurin pathway, is the role of Ca by itself sufficient since the vacuolar Ca transporter Pmr1 seems to play a major role in the induction of the supra high chitin synthesis. Any hypothesis for linking these Pmr1 and Yvc1 to chitin synthesis? It would be good also to see the "unpublished " data mentioned in l158 of the action of Ca+caspo+CFW. The effect of FK506 may be depending only on the concentration of the compound and it looks like only one concentration was tested.
Did the authors think about a putative sensor of the concentration of chitin in the cell wall?

In spite of the imperfections noticed, I still feel that this work should be published since the use of microfluidic systems is smart and may make scientists look at the effect of a drug or a change in the environment in a more kinetically approach. And this is an important development. Moreover, I also think that the analysis of the role of chitin synthesis in fungal death should be pursued and that the Gow's lab will answer many of the questions asked above. The fact that this ms lead to many unsolved questions is indeed for me a "+" since it may stimulate interesting cell wall research in the future.

Reviewer 2

Advance summary and potential significance to field

This manuscript asks how the chitin content of the cell wall is upregulated and maintained in response to cell wall stress. The major result is that the calcineurin pathway, which was previously shown to contribute to upregulation of chitin synthesis in response to cell wall stress, apparently also down-regulates chitin levels. In addition, calcineurin regulates chitin levels through the cell wall integrity pathway. The paper shows that calcium channels and upstream regulators of the calcineurin pathway do not modulate or attenuate cellular chitin levels. The experiments underlying these findings are a somewhat confusing because the two stresses being compared are not normalized for any specific factor and because of the multiple, potentially overlapping, roles of calcofluor white with calcium. Several major questions remain unaddressed, and some interpretations of the results need to be clarified.

Comments for the author

The revised manuscript from Dantas et al. is improved, however some serious concerns remain about the rationale of the study. The major concern is that two different types of treatments ((#1) caspofungin, a cidal antifungal drug and (#2) calcofluor white + Ca+2 (a stain for chitin that also stimulates additional chitin accumulation in the cell wall)) were used, each at a single concentration. The results were that #1 was more likely to yield extremely high chitin accumulation levels AND that #1 caused more cell death. However, since #1 is a cidal drug, the relationship between chitin accumulation and cell death is, at best, a correlation and does not demonstrate a causative relationship. Yet, the authors assume that this relationship is causative and much of the study is based upon this assumption.

A second issue is that several aspects of the study use calcofluor white and Ca+2 in multiple manners that makes it difficult to dissect what is causing what.
Calcofluor white + Ca+2 is used not only as a stress that is compared with caspofungin, but also as a pretreatment of cells prior to caspofungin treatment and as the stain used to visualize chitin accumulation in all cells. And, these authors and others have shown that calcium also induces calcineurin activity. In fact, prior work from Munro et al. 2007 shows that calcofluor white treatment alone results in increased expression of chitin synthases, and Walker et al. 2008 states

© 2021. Published by The Company of Biologists under the terms of the Creative Commons Attribution License (https://creativecommons.org/licenses/by/4.0/).

(but does not show) that calcofluor white pretreatment alone conferred some degree of protection from caspofungin. Here, the authors now argue that calcofluor white +Ca+2 reduces the supra-high chitin accumulation (as visualized by the calcofluor white) but the proposed model (supplementary figure 1) and the authors' response to Reviewer #2 comment #4 emphasize Ca+2 as a central component of this protection against caspofungin. Without separating the roles of these two chemicals, it is difficult to reach definitive conclusions.
In addition, a few comments about the responses to the prior review:
Comment 1 from Reviewer #2 was not a request for in vivo experiments, but rather for a simple in vitro experiment showing that strains that produce higher amounts of chitin also exhibit worse growth. This would provide strong evidence that the supra-high chitin levels seen with stresses #1 or #2 were the cause of death rather than an unrelated phenomenon. New supplementary figure 2 appears to show that supra-high chitin cells (except for those on the left, which appear to have been physically crushed between the 4- and 8-hour time points) can produce offspring after the stress is removed (especially cells on the right that are not indicated with an arrowhead). This suggests that supra-high chitin is not, in itself, lethal.
In response to Reviewer #2 major point 3 (as well as point 4 from Reviewer #1):
the authors show that both caspofungin and calcofluor white + Ca+2 can induce the formation of cells with supra-high chitin levels, albeit at different frequencies.
At the core of the work is the concept that Ca+2 induced activity of the calcineurin pathway reduces the cell wall integrity-pathway-mediated overproduction of chitin. The referenced Munro et al. 2007 shows a synergistic effect of calcofluor white with calcium chloride on the expression of chitin synthases but does not address the effect on viability.
To compare the role of calcineurin on chitin accumulation with the two stressors the authors would need to show that pretreatment with calcium protects against excess chitin accumulation using concentrations of caspofungin and calcofluor white which have an equivalent effect on chitin accumulation and viability.
Alternatively, they could pretreat with FK506 and monitor changes in chitin accumulation with the two stresses (again at concentrations that have equivalent effects in the absence of FK506).
It is also important to note that calcium has many roles beyond its impact on calcineurin (or its augmentation of calcofluor white), so such conclusions would need to be moderated by this caveat.

Second revision

Author response to reviewers' comments

Thank you for these further comments and suggestions for experiments that can be taken forward as we progress this study. Recognising the referees' suggestions for further clarification of the core message, we have undertaken more experiments (Table S1), made further alterations to the manuscript (blue highlights along with the yellow highlights from the original rebuttal) and now include a new Figure (Supplementary Fig. 1B) that serves as a summary of how chitin content correlates with stress resistance and viability. We have also modified several of the cartoons in Fig. 4, and Fig. S1A to take account of the further comments received and made minor changes to the Figure legends to improve clarity.

Table S1. Changes in chitin content and loss of viability in mutants of the calcineurin pathway after treatment of yeast cells with 0.2 M CaCl2 and 100 µg/mL CFW for 12 h. Values are SEMs (n=4 independent experiments). Significance compared to the wild-type strains * p≤0.05, ** p≤0.01

Strain	Loss off viability on Ca+CFW treatment (%)	Fold-change in chitin content (live cells)	Fold-change in chitin content (dead cells)
wt	3.9 ± 2.7	2.20 ± 0.20	21.32 ± 4.46
cna1Δ	33.18 ± 5.79**	9.74 ± 5.53*	14.36 ± 6.11
cnb1Δ	26.88 ± 11.84**	10.37 ± 4.13*	19.28 ± 13.58
crz1Δ	25.9 ± 8.27**	11.44 ± 5.02*	12.65 ± 3.57

© 2021. Published by The Company of Biologists under the terms of the Creative Commons Attribution License (https://creativecommons.org/licenses/by/4.0/).

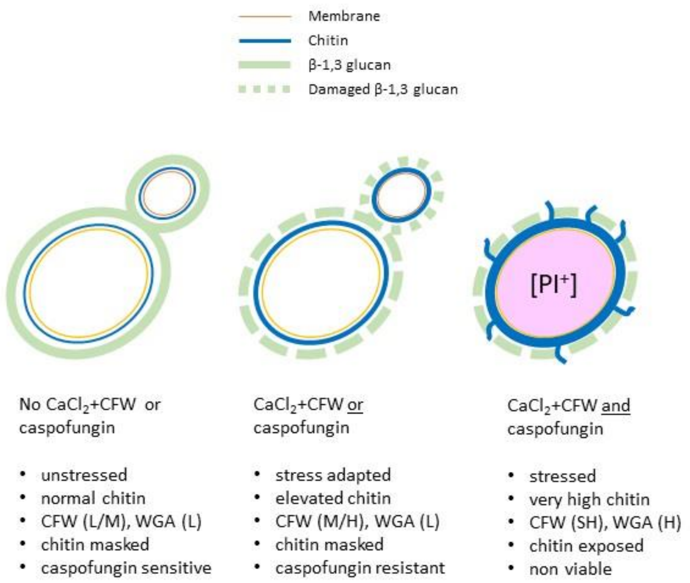

Supplementary Fig. 1B. Model based on data presented describing the relationship between chitin accumulation, sensitivity to caspofungin and cell viability. Intensity of CFW staining cells classified as low (L), medium (M), high (H) or supra-high (SH). WGA staining is normally masked by ß-1,3 glucan but occurs when chitin becomes exposed at the surface.

Reviewer 1

Advance Summary and Potential Significance to Field:

The use of a microfluidic system to better understand the effect of an antifungal drug is smart with a kinetic approach is smart and this is an important development. The analysis of the role of chitin synthesis in fungal death should be pursued and that the Gow's lab will answer many of the questions asked in this review. The fact that this ms lead to many unsolved questions is indeed for me a "+" since it may stimulate interesting cell wall research in the future.

Response: Thank you for our comments. Indeed, the study, presenting a new paradigm in the field, does present exciting opportunities for future work.

Reviewer 1 Comments for the Author: This reviewer thanks the authors for improving the clarity of the ms which unfortunately remains very puzzling; it is a very difficult piece of work to read and understand and I am still not totally convinced by the conclusions presented.

Response: We appreciate that there are some counter intuitive aspects of the results we present – for example in terms of chitin you can have too much of a good thing. We have made further revisions to the Abstract and the Introduction and added a summary Figure to further clarify the key messages (Supplementary Fig. 1B). Fundamentally, those cells with the highest chitin content (a 40-fold increase over untreated wild-type cells) are PI positive (less viable) (Fig. 1 C,D). We show this in multiple ways- by FACS, microfluidic time-lapse experiments and by microscopy. This is a novel observation that reveals a new cell wall homeostatic mechanism that may be fundamental in the fungi. We have made textual changes to clarify further.

The role of chitin here remains difficult to recognize: sometimes it is good for the fungus sometimes it is bad for growth and all this is only based on very little differences in the amount of chitin. In addition, the additional data giving a quantification of the amount of chitin in the cell wall remains imperfect since they are only relative: Ca+CFW gives a 0.7% increase in chitin / b1,3 glucan; caspo a 2.0% increase. But is it due to a reduction of b1,3 glucan consecutive to the caspo treatment rather than a true weight increase in chitin ie mg of chitin per mg of cell wall? The increase in chitin remains truly moderate and many fungi have a very high amount of chitin in their

© 2021. Published by The Company of Biologists under the terms of the Creative Commons Attribution License (https://creativecommons.org/licenses/by/4.0/).

cell wall without being perturbed in their growth. It even leads to a provocative comment. Are we sure that it is the chitin increase which is responsible for the growth inhibition or are we only looking at a secondary compensatory event due to an environmental stress which has nothing to do with the fungal death which could then be due to another cause? The authors suggest that the increase in chitin induced a modification of the elasticity of the cell wall which would be responsible for the death of the fungus but this “cell wall elasticity” is not quantified. Similarly, the authors speculate that the modification of the cell wall could be due to an alteration of the links between chitin and β-1,3 glucan but this is purely hypothetical since no chemical data are supporting this statement.

Response: We provided the data on cell wall analyses in two different ways. Supplementary Figure 4 shows both the percentage of mannan, glucan and chitin (Fig. S4A) as well as the absolute amounts (Fig. S4B). This shows that the absolute levels of the cell wall components and the percentage value both change. Chitin levels do increase a bit as a population average - but population averages disguise what we are able to identify using cell-sorting, which is a sub-population of supra-high chitin cells. We also need to underline the fact that, in cell wall physiology, more wall does not mean a stronger wall. For example, we showed previously that the thin cell walls of lactate-grown cells are in fact stiffer (they have a higher Young’s modulus) than the more elastic cell walls of glucose-grown cells (Ene *et al*., 2015). The fact remains that cells with greatly elevated chitin progressively lose viability.

The reviewer is right that we hypothesise that other types of cell wall reorganisation may be important – e.g. chitin-glucan cross-linking (lines 569-570 now 417- 418 in the original revised manuscript). It would seem highly appropriate to mention this speculation in the discussion. Undertaking a systemic analysis of all the possible changes in the cell wall is clearly beyond this publication.

We note in addition that previous papers using physical compression of the wall by micromanipulation as a measure of stiffness and fractional deformation at bursting suggested that chitinous bud scars make the cell wall stiffer (Touhami *et al*., 2003; Chaudhari *et al*., 2012) (lines 464-469). Nonetheless, it is fully acknowledged that the reviewer’s hypotheses can apply equally to these earlier publications.

The role of chitin synthase genes is not also clear especially when the authors write in their response to a reviewer that the upregulation of chitin synthase genes does not lead to an increase in chitin”. Similarly, the authors interpret a better WGA staining by an increase in chitin of the external layer of the cell wall. It may only be due to an increased permeability of the cell wall to WGA due to a modification of the permeability of the cell wall resulting from the deleterious effect of the antifungal drug. Could the UDP-GlcNAc synthesis pathway be stimulated by caspofungin and be the only one responsible for the chitin increase? For the calcineurin pathway, is the role of Ca by itself sufficient since the vacuolar Ca transporter Pmr1 seems to play a major role in the induction of the supra high chitin synthesis. Any hypothesis for linking these Pmr1 and Yvc1 to chitin synthesis? It would be good also to see the “unpublished “ data mentioned in l158 of the action of Ca+caspo+CFW. The effect of FK506 may be depending only on the concentration of the compound and it looks like only one concentration was tested. Did the authors think about a putative sensor of the concentration of chitin in the cell wall?

Response: The referee raises some interesting hypothetical questions- all of which could be incorporated into future studies of this new mechanism. WGA is a high molecular weight lectin and does not permeate to the inner wall. Permeability is determined mainly by the mannan content. It is therefore theoretically possible, but less likely, that WGA agglutinin is reporting a change in wall permeability rather than exposure of chitin on the wall outer surface. It is possible that the vacuolar store of calcium ions could be accessed and participate in the activation of the calcineurin- dependent suppression of the cell wall integrity pathway. We know from the results as presented that upstream calcium channels are apparently not required, but the role of calcium stores would require further investigation and we prefer not to over-speculate. Our model does not require identification of a chitin sensor per se. We are hypothesising as to what is the most plausible explanation - namely that it the flex and elasticity in the cell wall activates stress-dependent mechanisms. Nonetheless, we have acknowledged this in the discussion (lines 491-495) and added a hypothetical sensor in the overall summary cartoon (Supplementary Fig. 1A) to

© 2021. Published by The Company of Biologists under the terms of the Creative Commons Attribution License (https://creativecommons.org/licenses/by/4.0/).

acknowledge this possibility.

We know that the concentration of FK506 we used is effective because: (a) we see increased killing with $CaCl_2$ + CFW and FK506 and (b) we see higher chitin cells in the presence of FK506.

To further address this we have included data from new experiments that demonstrate that the level of chitin in three calcineurin pathway mutants - *cna1*, *cnb1* and *crz1* is elevated after $CaCl_2$ + CFW treatment and that this is accompanied by increased loss of viability (lines 394-398,& Table S1). In these mutants there is an increased chitin content compared to wild type in response to $CaCl_2$ + CFW and a significantly increased loss of viability. Again this supports the view that the calcineurin pathway acts to prevent over-activation of chitin synthesis and loss of viability.

In spite of the imperfections noticed, I still feel that this work should be published since the use of microfluidic systems is smart and may make scientists look at the effect of a drug or a change in the environment in a more kinetically approach. And this is an important development. Moreover, I also think that the analysis of the role of chitin synthesis in fungal death should be pursued and that the Gow's lab will answer many of the questions asked above. The fact that this ms lead to many unsolved questions is indeed for me a "+" since it may stimulate interesting cell wall research in the future.

Response: Thank you for these comments. We are indeed planning the next coherent set of experiments to further investigate this new observation.

Reviewer 2

Advance Summary and Potential Significance to Field: This manuscript asks how the chitin content of the cell wall is upregulated and maintained in response to cell wall stress. The major result is that the calcineurin pathway, which was previously shown to contribute to upregulation of chitin synthesis in response to cell wall stress, apparently also down-regulates chitin levels. In addition, calcineurin regulates chitin levels through the cell wall integrity pathway. The paper shows that calcium channels and upstream regulators of the calcineurin pathway do not modulate or attenuate cellular chitin levels. The experiments underlying these findings are a somewhat confusing because the two stresses being compared are not normalized for any specific factor and because of the multiple, potentially overlapping, roles of calcofluor white with calcium. Several major questions remain unaddressed, and some interpretations of the results need to be clarified.

Response: We agree that there are some counter-intuitive aspects of our findings and that the pathways that are involved can have multiple outputs. We have made further revisions to the manuscript and added a figure that summarises the ways in which chitin levels correlate with viability and sensitivity to caspofungin and a new data table (Table S1) with more evidence for the role of the calcineurin pathway encoding the chitin attenuation response.

Reviewer 2 Comments for the Author:

The revised manuscript from Dantas et al. is improved, however some serious concerns remain about the rationale of the study. The major concern is that two different types of treatments ((#1) caspofungin, a cidal antifungal drug and (#2) calcofluor white + Ca^{+2} (a stain for chitin that also stimulates additional chitin accumulation in the cell wall)) were used, each at a single concentration. The results were that #1 was more likely to yield extremely high chitin accumulation levels AND that #1 caused more cell death. However, since #1 is a cidal drug, the relationship between chitin accumulation and cell death is, at best, a correlation and does not demonstrate a causative relationship. Yet, the authors assume that this relationship is causative and much of the study is based upon this assumption.

Response: Firstly, the suggestion that caspofungin is "fungicidal" is a simplification. To be precise, "fungicidal" is actually the net population-wide effect of both cidal and non-cidal events. For example, it has been documented that, for *Aspergillus,* the initial lysis of most hyphal tips can be followed by adaptation and the formation of thicker, slow-growing fungal hyphae - even in the presence of caspofungin (Walker *et al*., 2015; Ries *et al*.,2017). This is relevant to the so-called "paradoxical growth effect" that has been shown for both *Aspergillus* and *Candida*. Similarly, we

© 2021. Published by The Company of Biologists under the terms of the Creative Commons Attribution License (https://creativecommons.org/licenses/by/4.0/).

showed that the MIC can be 7 doubling dilutions greater if chitin is moderately stimulated by pre-growing in $CaCl_2$ + CFW (Walker *et al.*, 2008, Fig. 4). It should also be noted that we have previously observed and published that high chitin, non-growing persistent cells in mouse tissue affect echinocandin tolerance (Lee *et al.*, 2012).

In our new figure (Supplementary Fig. 1B), we refer to these cells with moderately elevated chitin levels as “stress adapted”. What we show in this work is that a fraction of cells that are simultaneously exposed to multiple chitin-stimulating agonists accumulate a very high (pathological) level of chitin. Chitin is stimulated by $CaCl_2$ + CFW and by caspofungin. The referee is correct that this is a correlation and we have underlined this in the revised text (lines 45; 428; 449-450; 464-469).

A second issue is that several aspects of the study use calcofluor white and Ca+2 in multiple manners that makes it difficult to dissect what is causing what. Calcofluor white + Ca+2 is used not only as a stress that is compared with caspofungin, but also as a pretreatment of cells prior to caspofungin treatment and as the stain used to visualize chitin accumulation in all cells. And, these authors and others have shown that calcium also induces calcineurin activity. In fact, prior work from Munro et al. 2007 shows that calcofluor white treatment alone results in increased expression of chitin synthases, and Walker et al. 2008 states (but does not show) that calcofluor white pretreatment alone conferred some degree of protection from caspofungin. Here, the authors now argue that calcofluor white +Ca+2 reduces the supra-high chitin accumulation (as visualized by the calcofluor white) but the proposed model (supplementary figure 1) and the authors’ response to Reviewer #2 comment #4 emphasize Ca+2 as a central component of this protection against caspofungin. Without separating the roles of these two chemicals, it is difficult to reach definitive conclusions.

Response: The referee is correct that we are using $CaCl_2$ + CFW as a wall stress (as it has been used in multiple reports to date). The rationale of the study was to convey that cells with very high chitin content lose viability. We do not contest that multiple mechanisms may be at play, but it is clearly an advance to know that too much chitin may be detrimental to cell viability. Indeed, the project started with a hypothesis that there would be simple proportional relationship between chitin content and caspofungin resistance – but we found instead that the small fraction of cells with the highest chitin levels were dead or compromised in viability. Dissecting the roles of CFW and $CaCl_2$ on the transcriptional and post-transcriptional events that modify the cell wall will be an important but enormous task for the future and is yet to be achieved despite their use in many dozens of publications. See also the discussion above and data in the new Table S1.

In addition, a few comments about the responses to the prior review: Comment 1 from Reviewer #2 was not a request for in vivo experiments, but rather for a simple in vitro experiment showing that strains that produce higher amounts of chitin also exhibit worse growth. This would provide strong evidence that the supra-high chitin levels seen with stresses #1 or #2 were the cause of death, rather than an unrelated phenomenon. New supplementary figure 2 appears to show that supra-high chitin cells (except for those on the left, which appear to have been physically crushed between the 4- and 8-hour time points) can produce offspring after the stress is removed (especially cells on the right that are not indicated with an arrowhead). This suggests that supra-high chitin is not, in itself, lethal. In response to Reviewer #2 major point 3 (as well as point 4 from Reviewer #1): the authors show that both caspofungin and calcofluor white + Ca+2 can induce the formation of cells with supra-high chitin levels, albeit at different frequencies. At the core of the work is the concept that Ca+2 induced activity of the calcineurin pathway reduces the cell wall integrity-pathway-mediated overproduction of chitin. The referenced Munro et al. 2007 shows a synergistic effect of calcofluor white with calcium chloride on the expression of chitin synthases but does not address the effect on viability.

Response: To summarise, we claim the following: (a) both $CaCl_2$ + CFW and caspofungin induce chitin (live and fixed cells by microscopy, microfluidics, FACS and HPLC) and that (b) the low viability of caspofungin plus $CaCl_2$ + CFW treated cells correlate with the amount of supra-high chitin cells (high WGA, high ratio chitin/glucan, FACS data). Our data also support the role of the calcineurin pathway in modulating the chitin content in the wall. We have shown in our microfluidics experiments that the very high chitin cells are those that accumulate propidium

© 2021. Published by The Company of Biologists under the terms of the Creative Commons Attribution License (https://creativecommons.org/licenses/by/4.0/).

iodide, suggesting loss of viability. We also show in the new data (Table S1), that calcineurin mutants have elevated chitin and reduced viability in response to $CaCl_2$ + CFW.

To compare the role of calcineurin on chitin accumulation with the two stressors, the authors would need to show that pre-treatment with calcium protects against excess chitin accumulation using concentrations of caspofungin and calcofluor white which have an equivalent effect on chitin accumulation and viability. Alternatively, they could pretreat with FK506 and monitor changes in chitin accumulation with the two stresses (again at concentrations that have equivalent effects in the absence of FK506). It is also important to note that calcium has many roles beyond its impact on calcineurin (or its augmentation of calcofluor white), so such conclusions would need to be moderated by this caveat.

Response: Indeed the experiments presented in Figures 2 and 8 involve pre-treatment with $CaCl_2$ + CFW and this does indeed protect wild type cells on subsequent exposure to caspofungin. This was also shown in Walker et al (2008). We show that both *cna1*Δ and *crz1*Δ mutants display higher levels of chitin in response to $CaCl_2$ + CFW (Fig. 3A and Fig. 8A right hand side, top panel) as does *cna1*Δ in response to caspofungin (Fig. 8A - left hand side panel, bottom panel). Although we suggest that calcineurin acts as a negative regulator of chitin over-production (again see new Table S1), we acknowledge that an intact calcineurin pathway contributes to modest upregulation of chitin and hence resistance to caspofungin. Furthermore, we show that wild type cells treated with FK506 and treated with $CaCl_2$ + CFW display a 40% reduction in growth (Fig 6A) and increase in chitin levels, but not necessarily to supra high chitin cells (Fig 6B – high CFW but not WGA positive – Supplementary Fig. 1B). This is consistent with phenotypes associated with inhibition of calcineurin.

The referee requested a moderate statement based on the caveat that calcium has potentially multiple roles in which it could affect the phenotype. The discussion has been modified accordingly (lines 491-495).

References:

Chaudhari, R.D., Stenson, J.D., Obverton, T.W and Thomas, C.R. (2012). The effects of bud scars on the mechanical properties of *Saccharomyces cerevisiae* cell walls. Chemical Engineering Science 84 (2012) 188–196.

Ene, I.J., Walker, L.A., Schiavone, M., Lee, K.K., Martin-Yken, H., Dague, E. Gow, N.A.R., Munro, C.A. and Brown, A.P.J. (2015). Cell wall remodelling enzymes modulate fungal cell wall elasticity and osmotic stress resistance. *mBio*. 6(4), e00986-15. doi: 10.1128/mBio.00986-15.

Ries LNA, Rocha MC, de Castro PA, Silva-Rocha R, Silva RN, Freitas FZ, de Assis LJ, Bertolini MC, Malavazi I, and Goldman GH. 2017. The *Aspergillus fumigatus* CrzA transcription factor activates chitin synthase gene expression during the caspofungin paradoxical effect. mBio 8:e00705-17. https://doi.org/10.1128/mBio.00705-17

Touhami, A., Nysten, B. and Dufrene, Y.F. (2003). Nanoscale mapping of the elasticity of microbial cells by atomi and force microscopy. Langmuir 19, 4539–4543.
Walker, L.A., Lee, K.K., Munro, C.A. and Gow, N.A.R. (2015). Caspofungin treatment of *Aspergillus fumigatus* results in ChsG-dependent upregulation of chitin synthesis and the formation of chitin-rich micro-colonies. *Antimicrobial Agents and Chemotherapy* 59, 5932-5941.

Third decision letter

MS ID#: JOCES/2021/258889

MS TITLE: Crosstalk between calcineurin and the cell wall integrity pathways prevents chitin overexpression in Candida albicans

© 2021. Published by The Company of Biologists under the terms of the Creative Commons Attribution License (https://creativecommons.org/licenses/by/4.0/).

AUTHORS: Alessandra da Silva Dantas, Filomena Nogueira, Keunsook Lee, Louise Walker, Matt Edmondson, Alexandra Christine Brand, Megan D Lenardon, and Neil Andrew Robert Gow
ARTICLE TYPE: Research Article

I am happy to tell you that your manuscript has been accepted for publication in Journal of Cell Science, pending standard ethics checks.

© 2021. Published by The Company of Biologists under the terms of the Creative Commons Attribution License (https://creativecommons.org/licenses/by/4.0/).
